# Complement Signaling and Regulated Cell Death in Immune-Mediated Glomerular Injury

**DOI:** 10.3390/medicina62101924

**Published:** 2026-10-06

**Authors:** Eleni Tzali, Christos Pleros, Kostas Stylianou, Ioannis Petrakis

**Affiliations:** 1 Department of Nephrology, School of Medicine, University of Crete, 71003 Heraklion, Greece; lena.tzali@gmail.com (E.T.); kstylianu@uoc.gr (K.S.); 2 Department of Nephrology, Heraklion University Hospital, 71500 Crete, Greece; xpleros@gmail.com

**Keywords:** complement system, regulated cell death, glomerular diseases

## Abstract

The complement system is a central component of innate immunity, with essential roles in host defense, immune complex clearance, apoptotic cell removal, and tissue homeostasis. In the kidney, glomerular and tubular structures are continuously exposed to circulating and locally produced complement components. Dysregulated complement activation contributes to a wide spectrum of renal diseases, including membranous nephropathy, IgA nephropathy, ANCA-associated glomerulonephritis, lupus nephritis, thrombotic Imicroangiopathies and selected forms of podocyte injury. Regulated cell death pathways, including apoptosis, necroptosis, pyroptosis, ferroptosis, and autophagy, further shape renal inflammation and injury. These pathways interact bidirectionally with complement: complement effectors such as C3a, C5a, and the membrane attack complex can trigger or modulate inflammatory cell death, whereas dying cells release danger signals and altered-self structures that amplify complement activation. This crosstalk creates self-reinforcing loops of necroinflammation, glomerular injury, tubular damage, and fibrosis. This comprehensive review integrates current evidence on complement biology and regulated cell death in renal disease, emphasizing mechanistic links, disease-specific pathways, diagnostic implications, and emerging therapeutic opportunities. Understanding this interaction may support more precise approaches to complement inhibition, modulation of cell death, and personalized treatment in immune-mediated kidney disease.

## 1. Introduction

The complement system is an evolutionarily preserved component of innate immunity that serves as the first line of defense against pathogens and links innate to adaptive immunity [1,2]. It comprises a tightly regulated network of soluble plasma proteins, produced mainly by the liver, along with membrane-bound receptors and regulatory proteins expressed by numerous cell types. Collectively, these components mediate target recognition, opsonization, inflammation, immune cell recruitment, and membrane lysis. Complement is now recognized as a multifaceted immune system whose functions extend far beyond direct cytolysis [3,4].

The kidney’s glomerular filtration barrier is continuously exposed to circulating complement components under both normal and disease conditions. Renal cells also synthesize complement proteins locally and express related receptors and regulators, making the kidney both a target and a contributor to complement-mediated injury. Substantial evidence indicates that complement activation is a major pathogenic driver in several glomerular diseases, including membranous nephropathy, IgA nephropathy, ANCA-associated glomerulonephritis, and lupus nephritis [4]. Complement effectors can induce or modulate inflammatory signaling, cellular stress responses, and regulated cell death pathways, contributing to tissue injury even without overt complement-mediated lysis.

Regulated cell death (RCD) refers to controlled cell death mediated by defined molecular mechanisms that can, in principle, be modified through genetic or pharmacological intervention. Major modalities include apoptosis, necroptosis, pyroptosis, ferroptosis and, in specific contexts, autophagy-related cell death [5,6]. These processes differ in morphology, molecular machinery, and inflammatory consequences. Apoptosis is generally a non-lytic form of RCD, immunologically silent, involving caspase-mediated dismantling of the cell; necroptosis is a lytic form of regulated necrosis, mediated by receptor-interacting protein kinase 1/3 (RIPK1/RIPK3) and mixed lineage kinase domain-like protein (MLKL); pyroptosis is an inflammatory process, mediated by gasdermin D pore formation, frequently associated with inflammasome and inflammatory cytokine release; while ferroptosis is characterized by iron-dependent phospholipid peroxidation. Autophagy, by contrast, is primarily a homeostatic and stress-adaptive recycling process; autophagy-dependent cell death refers specifically to contexts in which the autophagic machinery contributes causally to cellular demise [5,6]. Beyond triggering or modulating regulated cell death, complement participates in bidirectional crosstalk in which dying or damaged cells expose altered-self structures and release danger signals that can further activate complement and amplify inflammation [7,8,9].

This comprehensive review integrates insights from complement biology and cell death research to elucidate mechanisms of renal disease. Part A provides an overview of complement in health and homeostasis, details the three activation pathways and their regulators, and describes the extracellular and intracellular functions of complement. Part B delves into the molecular features of regulated cell death modalities and their interactions with the complement system. Part C reviews the contribution of complement and regulated cell death to major glomerular diseases. The review concludes with clinical perspectives and future directions for therapeutic intervention.

## 2. Literature Review Methods

This article is a comprehensive narrative review. Relevant literature was identified through searches of PubMed/MEDLINE and Scopus conducted between January and July 2026. Search terms, individually and in combination, included “complement,” “complement system,” “glomerular disease,” “glomerulonephritis,” “cell death,” the various regulated cell death forms, and each glomerulonephritis mentioned in this review. Additional relevant publications were identified from the reference lists of key articles. Priority was given to primary mechanistic studies, human translational and clinical studies, major clinical trials, consensus or guideline documents, and recent high-quality reviews. Earlier seminal studies were also included where necessary to describe established biological mechanisms. Because this was a narrative rather than a systematic review, no formal meta-analysis or quantitative assessment of study quality was performed.

## 3. The Complement System

Beyond its role in host defense, the complement system contributes significantly to immune homeostasis. Under physiological conditions, complement facilitates clearance of immune complexes and apoptotic cells and participates in modulating adaptive immunity [1,3,7,8]. Efficient recognition of altered-self structures promotes macrophage phagocytosis via complement receptors, limiting the persistence of immunogenic cellular debris. Consistent with this homeostatic role, deficiencies of early components (e.g., C1q, C4) are strongly associated with systemic lupus erythematosus (SLE) [10].

**Figure 1:**
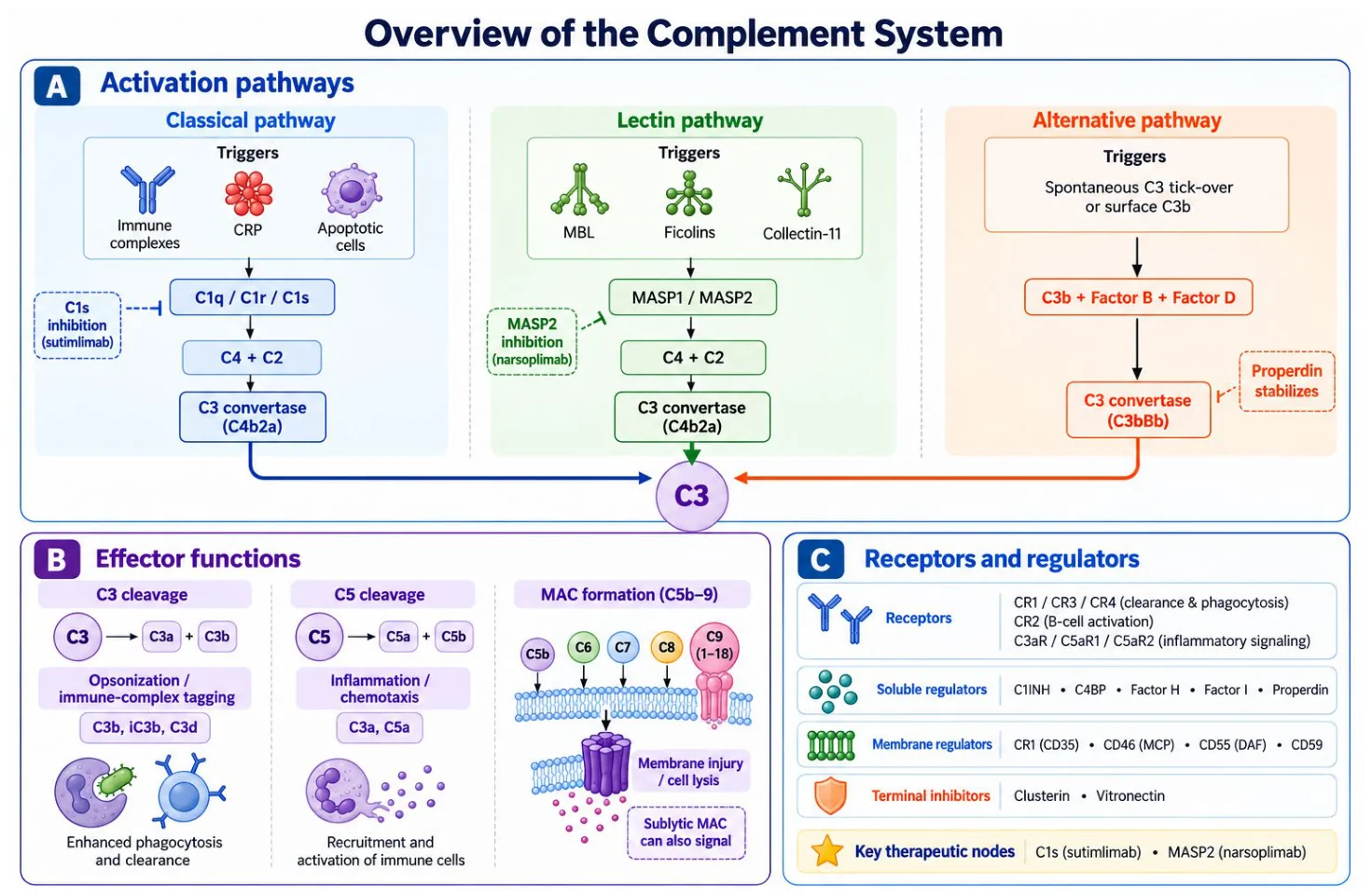
Overview of the complement system. (**A**) Complement activation pathways. (**B**) Terminal pathway and effector functions. (**C**) Complement receptors and therapeutic nodes. Schematic diagram only (AI-assisted illustration), no data-derived content. See Table 1, Table 2 and Table 3 and corresponding text sections for evidence details and references.

Complement activation proceeds through three major pathways—the classical, lectin, and alternative pathways—which converge at the level of C3 activation and subsequently engage common terminal effector mechanisms (Figure 1) [1,2,4].

Classical Pathway.

The classical pathway is initiated when C1q, a hexameric molecule, binds to the Fc region of antigen-bound IgM or IgG antibodies. C1q can also recognize non-immunoglobulin ligands, including C-reactive protein, structures exposed on apoptotic cells, and amyloid. Ligand binding activates the C1q-associated serine proteases C1r and C1s. Activated C1s cleaves C4 into C4a and C4b, allowing C4b to attach covalently to the target surface through its exposed thioester. C2 subsequently binds to C4b and is cleaved by C1s, generating the classical pathway C3 convertase, C4b2b. This convertase cleaves C3 into C3a and C3b, and incorporation of an additional C3b molecule generates the C5 convertase C4b2b3b, which cleaves C5 into C5a and C5b (Figure 1) [1,2].

Lectin Pathway.

The lectin pathway is activated by soluble pattern-recognition molecules that recognize carbohydrate moieties or other molecular patterns on pathogen or altered-self surfaces. Mannose-binding lectin (MBL) recognizes carbohydrate motifs containing mannose, fucose, and N-acetylglucosamine, whereas collectins (e.g., collectin 11) and ficolins (L-ficolin, H-ficolin, M-ficolin) recognize acetylated molecular patterns. These recognition molecules circulate in association with MBL-associated serine proteases (MASPs), principally MASP-1 and MASP-2, serine proteases analogous to C1r/C1s. Following target recognition, MASP activation leads to cleavage of C4 and C2, with MASP2 generating the C3 convertase, C4b2a. The lectin and classical pathways therefore differ primarily in their recognition mechanisms but converge on the same downstream C3 and C5 convertases (Figure 1) [1,2,4].

Alternative Pathway.

Unlike the classical and lectin pathways, the alternative pathway maintains continuous low-level activity through spontaneous hydrolysis (“tick-over”) of C3. C3 undergoes continuous low-grade hydrolysis, forming C3(H2O), which binds to soluble factor B. Factor D (also known as adipsin) cleaves factor B to generate Bb, forming the fluid-phase C3 convertase C3(H2O)Bb [1,2,11]. On activating surfaces, such as microbial surfaces or altered-self surfaces lacking complement regulators, C3b binds factor B; factor D cleaves B to generate the membrane-bound C3 convertase, C3bBb. Properdin stabilizes C3bBb and protects it from spontaneous decay. Because newly generated C3b can form additional C3 convertases, the alternative pathway also functions as an amplification loop for complement activation initiated through the classical or lectin pathways (Figure 1) [1,2,4,12].

Terminal Pathway and the Membrane Attack Complex.

Regardless of the initiating pathway, the complement cascade converges on the cleavage of C5 into C5a and C5b. C5b sequentially recruits C6 and C7, forming a complex (C5b-7) that inserts into the lipid bilayer of the target membrane. Binding of C8 initiates membrane penetration and pore formation. Multiple C9 molecules polymerize to form the membrane attack complex (MAC; C5b-9), a transmembrane channel. MAC formation leads to osmotic lysis of susceptible cells. Nucleated cells, however, can resist or recover from complement-mediated lysis by shedding MAC, recruiting complement inhibitors, and repairing membrane lesions (Figure 1). At sublytic densities, MAC can, instead, function as a signaling platform, activating intracellular pathways and modulating gene expression [13,14,15].

### 3.1. Complement Receptors and Regulators

**Figure 2:**
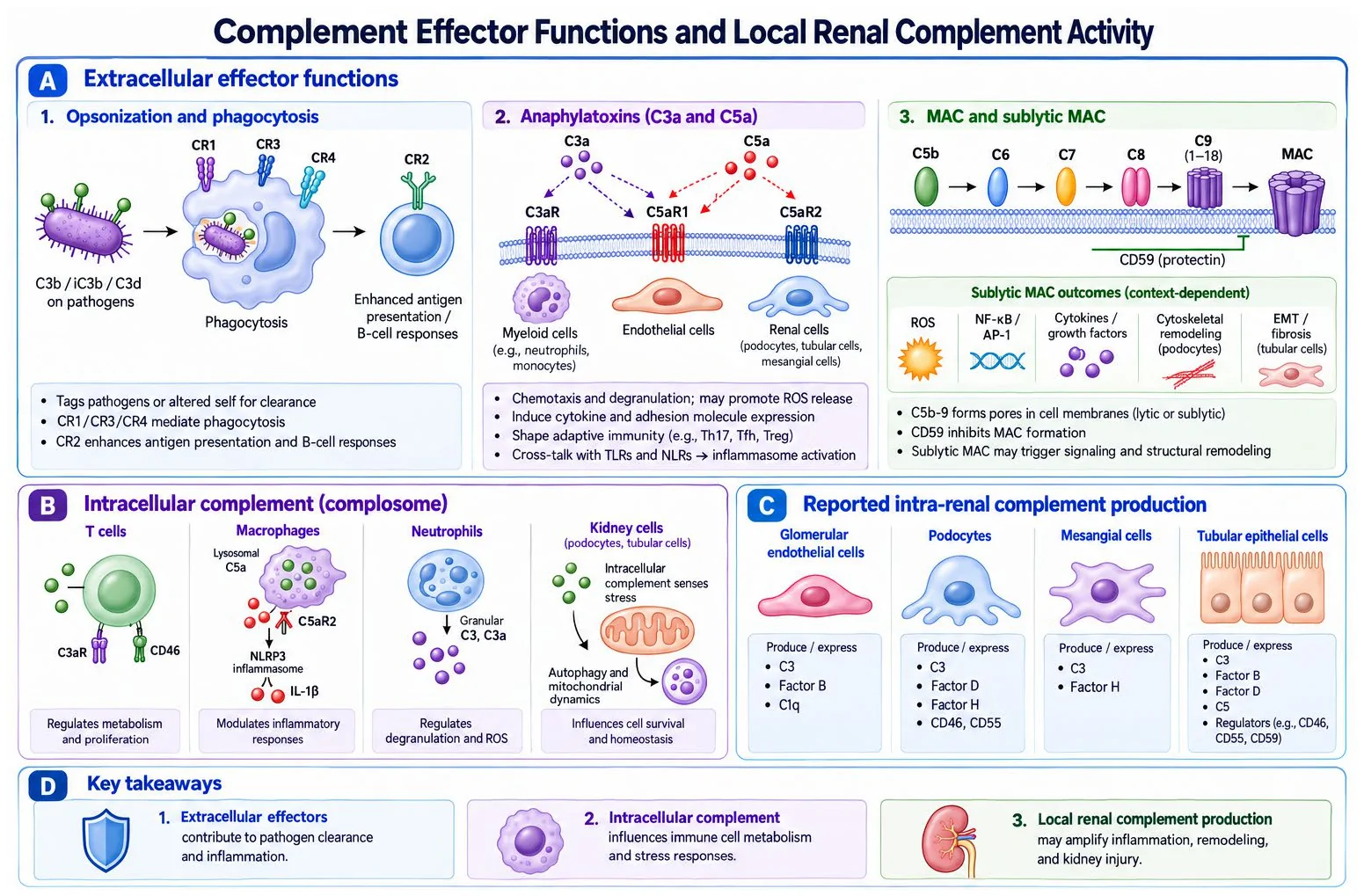
Extracellular and intracellular complement effector functions. (**A**) Extracellular effector functions: opsonization and phagocytosis, anaphylatoxins, and MAC and sublytic MAC signaling. (**B**) Intracellular complement (complosome). (**C**) Reported intrarenal complement production. (**D**) Key takeaways. Schematic diagram only (AI-assisted illustration), no data-derived content. See Table 1, Table 2 and Table 3 and corresponding text sections for evidence details and references.

Complement receptors translate complement activation into cellular responses, whereas soluble and membrane-bound regulators restrict complement amplification and protect host surfaces from inappropriate injury (Figure 1 and Figure 2). Together, these systems determine both the magnitude and the cellular consequences of complement activation.

CR1 (CD35) is a large transmembrane glycoprotein composed of multiple short consensus repeats (SCRs). It is expressed on erythrocytes, leukocytes, podocytes, and glomerular endothelial cells [16]. CR1 serves as a cofactor for factor I-mediated cleavage of C3b and C4b and accelerates decay of C3/C5 convertases, thereby limiting complement activation [7]. On erythrocytes, CR1 binds the complement-opsonized immune complexes, facilitating their transport to the liver and spleen for clearance. Mutations in CR1 or reduced CR1 expression are associated with susceptibility to systemic lupus erythematosus and immune complex glomerulonephritis [16].

CR2 (CD21) is a membrane protein expressed on B cells and follicular dendritic cells [9]. It binds C3-derived fragments (C3b, iC3b, C3d), forming a co-receptor complex with CD19 and CD81 [17,18]. CR2 engagement lowers the threshold for B-cell receptor activation and enhances antibody responses [18].

CR3 (Mac-1, CD11b/CD18) and CR4 (CD11c/CD18) are β2 integrins expressed predominantly on myeloid cells. They recognize iC3b, fibrinogen, and ICAM-1 and participate in adhesion, phagocytosis, migration, and related inflammatory functions. CR3 is upregulated on activated neutrophils and macrophages during inflammation. In ANCA-associated vasculitis, CD11b/CD18-dependent interactions contribute to neutrophil adhesion to activated endothelium and endothelial injury (Figure 1 and Figure 2). CR3 also interacts with complement-coated apoptotic cells to promote clearance and resolution of inflammation, whereas CR4 has overlapping roles in macrophages and dendritic cells [19,20].

CRIg, a member of the immunoglobulin superfamily, expressed on tissue-resident macrophages, such as Kupffer cells in the liver, binds C3b and iC3b and contributes to the clearance of circulating complement-opsonized pathogens (Figure 1 and Figure 2). However, the kidney-specific evidence is less extensive than for liver Kupffer cells [21].

C3aR and C5aR1 are seven-transmembrane G protein-coupled receptors, expressed on leukocytes and several renal parenchymal cell populations, including tubular epithelial cells. Through C3a and C5a binding, respectively, they mediate chemotactic, inflammatory, and cellular stress responses (Figure 1 and Figure 2). C5aR2 (C5L2), structurally related to C5aR1, lacks conventional G-protein coupling and has context-dependent function. Initially regarded predominantly as a decoy receptor, experimental studies now indicate that it can either restrain or amplify inflammatory signaling through interactions with C5aR1 and β-arrestin-dependent pathways. This means that the biological effect of C5a is determined not only by C5aR1 activation but also by the balance between C5aR1 and C5aR2 signaling [22].

Complement activation is controlled at multiple levels by soluble and membrane-associated regulators. C1 esterase inhibitor (C1INH) inhibits C1r, C1s, MASP-1, and MASP-2; its deficiency causes hereditary or acquired bradykinin-mediated angioedema through dysregulated activation of the plasma kallikrein–kinin pathway and excessive bradykinin generation. Factor H and factor I are major regulators of the alternative pathway. Factor I cleaves C3b and C4b in the presence of cofactors such as factor H, CR1, CD46, and C4BP; C4BP regulates classical/lectin pathway convertases; properdin stabilizes the alternative pathway C3 convertase; CD46 acts as a factor I cofactor. On host-cell membranes, CR1, CD46, and CD55 limit convertase activity, while CD59 inhibits terminal complement assembly by preventing C9 incorporation into the MAC; clusterin/vitronectin bind soluble terminal complement complexes [2,23].

Factor H is the principal soluble regulator of alternative pathway amplification. Its N-terminal domains provide factor I cofactor activity and accelerate decay of the C3bBb convertase (binding to C3b and facilitating factor I-mediated cleavage to iC3b). Its C-terminal domains recognize C3b and host-surface polyanions, such as glycosaminoglycans, thereby directing complement regulation preferentially to self surfaces [8,23,24]. Factor H-related proteins lack the N-terminal regulatory domains of factor H and can, in specific contexts, promote alternative pathway activation by competing with factor H for surface ligands, including C3b [25,26].

Genetic or acquired defects in alternative pathway regulation are central to kidney diseases such as complement-mediated thrombotic microangiopathy and C3 glomerulopathy. Pathogenic variants in CFH, CFI, CD46, C3, CFB, and the CFHR gene family can impair surface complement regulation or enhance alternative pathway amplification, although penetrance and clinical expression vary. Properdin deficiency predisposes to meningococcal infection, while alternative pathway dysregulation contributes to complement-mediated kidney injury and is a therapeutic target [2,12,27].

More broadly, in T cells, CD46 engagement by locally generated C3b contributes to intracellular complement signaling and the metabolic reprogramming required for Th1 responses [28]. In human T cells, locally generated C3 fragments can bind CD46 and provide a costimulatory signal that supports Th1 differentiation. CD46 signaling promotes the metabolic changes required for effector T-cell function, including increased nutrient uptake, glycolysis, mitochondrial activity, and mTOR activation, thereby enabling IFN-γ production and a sustained Th1 response [28]. CD59 inhibits MAC formation by preventing C9 polymerization, and glycation of CD59 in diabetes can reduce its complement-inhibitory function and enhance complement-mediated injury [29,30].

### 3.2. Extracellular Complement: Effector Functions

**Opsonization:** Covalent deposition of C3b and its degradation fragments, iC3b, and C3d, on pathogens or altered-self surfaces tags them for phagocytosis. Phagocytes expressing complement receptors CR1, CR3, and CR4 bind opsonized particles and internalize them. Opsonization also enhances antigen presentation by dendritic cells and B cell responses via CR2 (Figure 2) [1,2]. Severe C3 deficiency impairs opsonization and is associated with recurrent bacterial infections [27].

**Anaphylatoxins:** C3a and C5a are powerful inflammatory mediators acting through C3aR and C5aR1, respectively. These receptors are widely expressed on myeloid, endothelial, and renal parenchymal cells, including tubular epithelial cells [22,31,32]. Experimental renal studies demonstrate that anaphylatoxin signaling induces chemotaxis, degranulation, oxidative burst, and cytokine production. In addition to classical immune cells, renal tubular epithelial cells respond to C3a and C5a by producing chemokines, adhesion molecules, and extracellular matrix proteins (Figure 2). Anaphylatoxins also influence adaptive immunity: C3a promotes Th17 differentiation, whereas C5a can skew T cell responses toward Th1 or Treg depending on context. C3a and C5a crosstalk with toll-like receptors and NOD-like receptors (NLR) to modulate inflammasome activation, thereby influencing pyroptosis [22,31,32,33].

**MAC and Sublytic Signaling:** The MAC is traditionally viewed as a pore-forming lytic complex. Structural studies show that C5b6 recruits C7, exposing hydrophobic domains that permit their insertion into membrane lipid bilayers. C8 associates with C5b-7 and initiates pore formation, followed by polymerization of several C9 molecules to complete the transmembrane complex. CD59 limits MAC assembly by interacting with C8 and C9 and preventing C9 polymerization [13,23,30]. Nucleated cells, however, often survive limited MAC attack through mechanisms that include endocytosis, membrane shedding, and membrane repair. MAC deposition below the threshold required for overt lysis is therefore referred to as sublytic.

Sublytic MAC can act as a signaling stimulus rather than simply causing cell death. Experimental studies have shown activation of tyrosine kinases, phospholipase C, intracellular Ca^2+^ fluxes, reactive oxygen species, and transcriptional pathways including NF-κB and AP-1 (Figure 2). These signals promote cell proliferation, secretion of growth factors (e.g., PDGF, TGF-β) and proinflammatory cytokines (IL-1β, IL-6, IL-8), contributing to tissue remodeling. Thus, sublytic MAC triggers responses that are either cytoprotective, like heat-shock proteins or induction of autophagy, or cell-deleterious, like activation of the NLRP3 inflammasome [13,14,15,34,35].

In podocytes, sublytic MAC can induce actin cytoskeleton reorganization, resulting in foot process effacement and proteinuria [36,37,38,39]. While podocytes have limited regenerative capacity, persistent MAC deposition may disrupt cytoskeletal and slit diaphragm integrity and, when adaptive autophagy is insufficient, promote apoptosis or inflammasome-associated forms of cell death, as experimental models of complement-mediated glomerular injury demonstrate [34,40,41]. In membranous nephropathy specifically, complement-induced podocyte pyroptosis has also been demonstrated in experimental and human tissue studies, with mitochondrial dysfunction and inflammasome activation implicated in this process [42]. In endothelial cells, experimental studies indicate that sublytic MAC can promote activation, proinflammatory and prothrombotic signaling, and altered cell-cycle regulation, with either proliferative activation or arrest and apoptosis, depending on injury intensity and context. Thus, MAC is a potent modulator of cell behavior beyond its lytic role [13,14,15,43].

Table 1 summarizes the disease-specific consequences of these extracellular complement effector mechanisms and their proposed relationships with regulated cell death pathways.

**Table 1:** Complement activation and regulated cell death across renal diseases. H-O, human observational, biopsy, biomarker, or genetic evidence; H-T, human interventional/clinical trial evidence; A, animal-model evidence; V, in vitro or ex vivo evidence.

Disease	Dominant Pathogenic Trigger	Main Complement Pathway/Effectors	Characteristic Renal/Biopsy Pattern	Regulated Cell Death Links	Therapeutic Implication
Membranous nephropathy (MN)	Podocyte-directed immune complexes, most commonly PLA2R-associated autoantibodies [H-O]	Lectin and alternative pathway activation/amplification are strongly implicated; classical pathway participation, C3, C3a/C5a and C5b-9/MAC are detected in human disease [H-O], with mechanistic support from experimental systems [A, V].	Subepithelial electron-dense deposits, foot process effacement, and granular capillary-wall IgG, C3 and C5b-9; IgG4 predominance is characteristic of PLA2R-associated disease [H-O]	Sublytic MAC induces podocyte cytoskeletal and mitochondrial injury and apoptosis [A, V]. Pyroptotic signaling, impaired autophagy and ferroptosis-related pathways have mainly been demonstrated experimentally, with more limited human tissue evidence [H-O, A, V].	B-cell-directed therapy is clinically established [H-T]. Complement inhibition is mechanistically plausible but remains investigational in most MN settings [A, V; limited H-T].
IgA nephropathy (IgAN)	Galactose-deficient IgA1-containing immune complexes with mesangial deposition [H-O]	Lectin pathway activation involving MBL/MASP2/ficolins and alternative pathway amplification; mesangial C3 and terminal complement components are documented in human biopsies [H-O], supported by experimental studies [A, V].	Mesangial IgA-dominant deposits, frequently accompanied by C3, with mesangial proliferation and matrix expansion [H-O]	Complement-mediated mesangial and podocyte injury is supported by human pathology [H-O]. Sublytic MAC-mediated proliferation/apoptosis, inflammasome activation and pyroptosis are predominantly supported by experimental models [A, V].	Supportive and disease-modifying therapy remains central [H-T]. Complement-directed approaches, including factor-B inhibition, have entered clinical development/therapy, whereas other targets such as MASP2 remain pathway-specific investigational strategies [H-T].
ANCA-associated GN (ANCA-GN/AAV)	MPO- or PR3-ANCA-mediated neutrophil activation with NET formation [H-O, V]	Alternative pathway amplification and the C5a–C5aR1 axis are supported by human biomarker/tissue observations and extensive experimental evidence [H-O, A, V].	Pauci-immune necrotizing and crescentic GN, with variable focal deposition of complement components despite minimal immunoglobulin staining [H-O]	Neutrophil activation, NET formation and necroinflammatory endothelial injury are well supported [H-O, A, V]. Specific attribution to endothelial necroptosis or pyroptosis is based mainly on experimental evidence [A, V; limited H-O].	Avacopan was previously supported by clinical trial evidence and authorized in the EU; following retraction of the pivotal ADVOCATE publication and revocation of its EU marketing authorization, its efficacy evidence is now uncertain. Other complement-directed approaches remain investigational [H-T].
Lupus nephritis (LN)	Autoantibody-containing immune complexes directed against nuclear and other autoantigens [H-O]	Classical pathway activation is prominent, with C1q, C4, C3 and terminal complement deposition; alternative and lectin pathways may also contribute [H-O, A].	“Full-house” immunofluorescence; proliferative lesions in class III/IV, subepithelial deposits in class V, and variable tubulointerstitial complement activation [H-O]	Defective apoptotic-cell clearance is strongly implicated in SLE pathogenesis [H-O, A]. Pyroptosis, including gasdermin-related signaling, has human biopsy support in selected cell populations, whereas mechanistic links involving mitochondrial DNA, necroptosis, ferroptosis and autophagy are predominantly experimental [H-O, A, V].	Standard immunosuppression and targeted immune therapies remain central [H-T]. Complement inhibition and direct modulation of regulated cell death pathways remain investigational or adjunctive concepts.
Thrombotic microangiopathy/complement-mediated aHUS	Genetic or acquired dysregulation of alternative pathway control [H-O]	Alternative pathway overactivation with uncontrolled C3-convertase amplification, C5 activation and MAC formation [H-O, A, V].	Endothelial swelling, fibrin/platelet thrombi, mesangiolysis, double contours and arteriolar/arterial TMA lesions [H-O]	Complement-mediated endothelial injury and prothrombotic transformation are established [H-O, A, V]. Necroptotic and pyroptotic mechanisms are supported primarily by experimental endothelial models [A, V].	C5 inhibition is clinically established in complement-mediated TMA/aHUS [H-T]; genetic and acquired complement evaluation has direct clinical relevance [H-O].
Anti-GBM disease	Autoantibodies against the α3 chain of type IV collagen in glomerular and alveolar basement membranes [H-O]	Classical pathway activation with downstream C3/C5 activation and terminal complement formation [H-O, A].	Linear GBM IgG staining, necrotizing crescentic GN and variable C3 deposition [H-O]	Complement-dependent neutrophil recruitment and necroinflammatory glomerular injury are supported by human pathology and experimental models [H-O, A]. Evidence for specific regulated cell death pathways remains limited and predominantly experimental [A, V].	Plasma exchange and immunosuppression remain standard [H-T]. Complement inhibition has predominantly case-based or investigational relevance [limited H-O/H-T].
Infection-related GN (IRGN)	Microbial antigens and infection-associated immune complex formation [H-O]	Classical and alternative pathway activation with prominent C3 deposition and complement consumption; terminal pathway activation may also occur [H-O].	Exudative GN, C3-dominant or codominant staining, and subepithelial “hump-like” deposits in post-infectious forms [H-O]	Complement-amplified glomerular inflammation and secondary tubular injury are well recognized [H-O], whereas assignment to specific regulated cell death pathways is largely inferential or experimental [A, V].	Treatment of the underlying infection and supportive care are standard [H-T/H-O]. Persistent complement abnormalities may prompt evaluation for underlying complement dysregulation [H-O].
Focal segmental glomerulosclerosis (FSGS)	Heterogeneous podocyte injury resulting from primary, genetic, adaptive or secondary mechanisms [H-O]	Complement is not a universal pathogenic driver. C3, C4d and other complement components have been reported in subsets and in sclerotic lesions [H-O], with mechanistic evidence for complement-mediated podocyte injury derived mainly from experimental studies [A, V].	Segmental sclerosis, podocyte foot process effacement and progressive podocyte loss; complement deposition may occur in selected lesions [H-O]	Podocyte apoptosis is supported by human and experimental observations [H-O, A, V]. Pyroptosis, ferroptosis and autophagy dysregulation are predominantly supported by cellular and animal models [A, V].	Complement inhibition is not standard therapy [H-T] and should currently be considered experimental or potentially relevant only to mechanistically defined subsets.

### 3.3. Intra-Renal Complement Production

Local synthesis of complement components by renal cells has been demonstrated at both the transcript and protein levels [44,45,46,47,48,49,50,51,52,53,54]. Single-cell transcriptomic analyses reveal that glomerular endothelial cells express high levels of C3, factor B, and C1q (Figure 2). Podocytes produce functional C3 and factor H, while also expressing complement receptors and regulators, including CR1, CD46, and CD55, and upregulate complement genes in response to injury [44]. Mesangial cells synthesize C3 and factor H and express membrane regulators [45,46,47,48]. Proximal tubular epithelial cells represent another important source of locally produced complement and can synthesize components including C3, C4, and factor B [49,50,51].

Its synthesis can be enhanced by immune injury, proteinuria, cytokines, hypoxia, and metabolic stress, particularly in tubular epithelial cells but also in glomerular endothelial cells, podocytes, and mesangial cells [51,52,53,54,55]. Collectively, these findings indicate that locally produced complement is not merely a reflection of circulating complement, but can actively contribute to renal inflammation, injury, fibrosis, and repair [56]. These locally generated proteins may amplify C3 activation, generate C3a and C5a, recruit inflammatory cells, and promote tubular injury, endothelial dysfunction, podocyte stress, and fibrosis. Therefore, urinary complement should be interpreted as an integrated marker of local synthesis, activation, and leakage, with adjustment for proteinuria, hematuria, kidney function, and sample handling [55,56].

## 4. Regulated Cell Death Pathways in Renal Injury: Mechanistic Overview

In kidney disease, the balance and interplay among cell death pathways determine the extent of tissue injury and the nature of the inflammatory milieu [5,6]. Part B provides a mechanistic overview of regulated cell death pathways relevant to renal injury rather than a comprehensive disease-by-disease review. Evidence from acute kidney injury, diabetic kidney disease, crystal-associated nephropathy, transplant-related injury, and other renal conditions is therefore included selectively when it helps define the molecular mechanisms, detection, or experimental modulation of apoptosis, necroptosis, pyroptosis, ferroptosis, or autophagy.

### 4.1. Apoptosis

**Figure 3:**
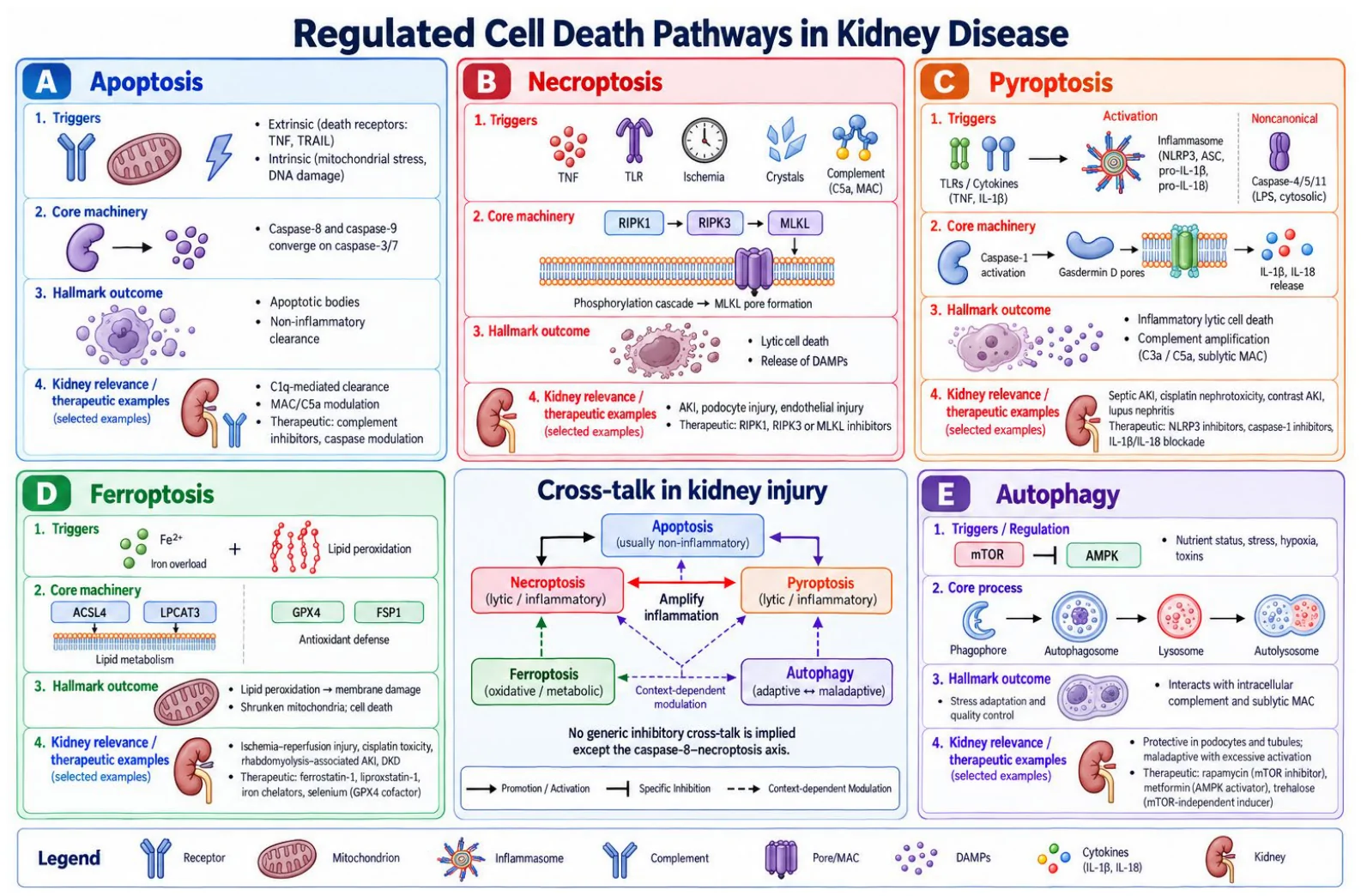
Regulated cell death in the kidney. Insights from pathophysiology and physiology. Schematic diagram only (AI-assisted illustration), no data-derived content. See Table 1, Table 2 and Table 3 and corresponding text sections for evidence details and references.

Apoptosis proceeds through two principal, converging pathways (Figure 3). The extrinsic pathway is initiated by extracellular death ligands engaging transmembrane death receptors (including Fas, TNF receptor 1, and TRAIL receptor). This assembly primarily leads to formation of the death-inducing signaling complex (DISC) and activation of initiator caspase-8, which in turn activates downstream effector caspases (caspases 3, 6, and 7). The intrinsic pathway is triggered by intracellular stress, such as DNA damage, oxidative stress, or growth factor deprivation. Mitochondrial outer membrane permeabilization occurs through pro-apoptotic Bcl-2 family members (including Bax and Bak) and results in cytochrome-c release, apoptosome formation, and caspase-9 activation. Both pathways converge on effector caspases, particularly caspases-3 and -7, producing characteristic features including chromatin condensation, nuclear fragmentation, and formation of apoptotic bodies. These bodies expose phosphatidylserine on their surface, serving as an “eat-me” signal to phagocytes and facilitating recognition and removal [5,6].

Apoptosis is generally non-lytic, and when apoptotic cells are efficiently cleared, it is also considered non-inflammatory because cellular contents remain sequestered. Complement contributes to the clearance process: C1q binds to apoptotic cells, promoting their uptake by macrophages and limiting cellular debris and secondary necrosis [10]. However, in pathological settings, impaired clearance of apoptotic bodies leads to secondary necrosis, the release of nuclear antigens, and complement activation. Deficiency of early classical pathway components, particularly C1q and C4, or receptor polymorphisms is strongly associated with and has been linked experimentally to accumulation of apoptotic material, providing a mechanistic connection between autoantigen exposure and loss of tolerance, in cases like systemic lupus erythematosus [10].

Complement effector molecules can also modulate apoptosis directly and bidirectionally. High density of MAC has been shown experimentally to trigger mitochondrial permeabilization and caspase activation, inducing apoptosis, whereas sublytic C5b-9 can promote mesangial cell apoptosis in experimental Thy-1 nephritis [57,58]. Conversely, in other experimental cell systems, sublytic C5b-9 activates PI3K/Akt-dependent survival pathways and can protect cells from apoptosis [35,59].

Experimental strategies to modulate apoptosis in kidney injury have included caspase inhibition and manipulation of Bcl-2-family signaling. However, their therapeutic effects are context-dependent, and inhibition of apoptosis does not necessarily translate into tissue protection because of crosstalk with other stress and cell death pathways [60].

### 4.2. Necroptosis

Necroptosis is a lytic form of regulated cell death mediated by RIPK3 and the executioner pseudokinase mixed lineage kinase domain-like protein (MLKL) (Figure 3). Necroptosis can function as an alternative programmed cell death pathway when apoptosis fails. In canonical TNF-induced necroptosis, inhibition or insufficient activity of caspase-8 permits RIPK1 kinase-dependent assembly of a RIPK1–RIPK3 signaling complex, commonly termed the necrosome. RIPK3 subsequently phosphorylates MLKL, inducing its oligomerization and translocation to the plasma membrane, where it disrupts membrane integrity and ion homeostasis, ultimately leading to cellular swelling and membrane lysis [61]. MLKL pores differ from MAC pores [62]; MLKL permeabilizes membranes from within, whereas MAC inserts from the extracellular space [63]. Necroptosis is inherently proinflammatory because it leads to the release of DAMPs and cytokines [64].

Necroptosis interacts with other cell death pathways [65]. Necrostatin-1, an experimental RIPK1 inhibitor widely used to block necroptosis, has been reported in some cellular models to also suppress ferroptosis-associated lipid peroxidation; however, this anti-ferroptotic effect may occur independently of RIPK1 inhibition [66,67]. Conversely, caspase-8 activity suppresses necroptosis by proteolytic regulation of RIPK1/RIPK3, although recent work indicates that direct cleavage of RIPK3 is not itself required for inhibition of necroptosis [68,69]. Necroptotic signaling can also intersect with inflammasome activation and pyroptosis, providing a mechanistic basis for amplification (release of DAMPS and mediators) of inflammatory cell death, termed “necroinflammation” [70,71].

In the kidney, experimental and human evidence implicates necroptosis particularly in tubular epithelial injury following ischemia–reperfusion and in crystal-associated nephropathy, while activation of the RIPK1–RIPK3–MLKL pathway has also been demonstrated in tubular epithelial cells in diabetic kidney disease. In autoimmune vasculitis, necroptosis regulates NET formation and contributes to complement activation and endothelial injury [70,71,72,73,74,75]. Renal necroptosis is triggered by TNF-α, TLR activation, ischemia–reperfusion, crystals (e.g., calcium oxalate) [71,76]. Experimental studies outside the kidney indicate that sublytic complement can engage RIPK1–RIPK3–MLKL signaling, suggesting a potential link between complement activation and necroptosis; however, this mechanism has not yet been directly established in renal tissue [77].

Demonstration of necroptosis includes increased expression of RIPK3 and phospho-MLKL, findings by themselves not sufficient. Stronger evidence provides morphological features of oncosis (such as cellular swelling and membrane ruptures) along with sensitivity to necrostatins and MLKL inhibitors [78,79]. Therapeutic candidates include RIPK1 inhibitors such as necrostatins, RIPK3 inhibitors such as dabrafenib, and MLKL inhibitors such as necrosulfonamide [80,81,82]. Preclinical murine studies suggest that pharmacological inhibition of RIPK1 or RIPK3 can attenuate renal injury in models of ischemic AKI, cisplatin nephrotoxicity, and calcium oxalate nephropathy [83,84,85].

### 4.3. Pyroptosis

Pyroptosis is an inflammatory form of regulated cell death that is primarily driven by inflammasome activation and gasdermin-mediated membrane pore formation. Inflammasomes are multiprotein complexes that sense danger signals and activate inflammatory caspases. NLRP3 inflammasome activation usually requires two steps: a priming signal, induced by toll-like receptors or proinflammatory cytokines, which activates NF-κB and increases the transcription of NLRP3 and pro-IL-1β; and an activation signal, triggered by cellular stressors such as potassium efflux, ionic fluxes, mitochondrial reactive oxygen species, or lysosomal damage (Figure 3). Pyroptosis is classified into canonical and non-canonical pathways.

In the canonical pathway, activated caspase-1 processes pro-IL-1β and pro-IL-18 into their mature forms and cleaves gasdermin D, releasing its pore-forming N-terminal fragment [86,87,88]. Gasdermin D N-terminal domains oligomerize at the plasma membrane to form approximately 10–15 nm pores, causing ionic fluxes, including K^+^ efflux, osmotic swelling, and eventual pyroptotic lysis [89,90]. This process promotes the release of inflammatory cytokines and cytosolic danger-associated molecular patterns, including HMGB1 (high-mobility group box 1), depending on the cellular context and the extent of membrane rupture [91,92]. In the non-canonical pathway, caspase-11 can cleave pannexin-1, leading to ATP release, P2X7 receptor activation, K^+^ efflux, and amplification of inflammatory signaling [93]. Conversely, ESCRT-III (endosomal sorting complex required for transport III)-dependent membrane repair can counteract gasdermin-D-mediated membrane damage and delay or limit pyroptotic lysis [94].

In the non-canonical pathway, cytosolic lipopolysaccharide directly activates murine caspase-11 or human caspases-4 and -5, which cleave GSDMD and can secondarily activate the NLRP3 inflammasome. Caspase-11-dependent pannexin-1 cleavage, ATP release, and P2X7 receptor activation have also been described as additional amplifying mechanisms, although GSDMD cleavage represents the core execution step of non-canonical pyroptosis [89,95]. Caspase-3 can also cleave gasdermin E, thereby converting apoptotic signaling into secondary pyroptosis in GSDME-expressing cells, in the absence of GSDMD [96,97].

In the kidney, experimental evidence implicates pyroptosis in cisplatin-induced nephrotoxicity, contrast-induced AKI, and immune-mediated glomerular diseases such as lupus nephritis [98,99,100]. In these settings, activation of NLRP3/caspase-1 or non-canonical caspase-4/5/11 pathways promotes gasdermin-mediated renal epithelial injury and inflammatory cytokine release. Complement activation may further amplify pyroptotic signaling. In membranous nephropathy, complement-induced podocyte pyroptosis has been demonstrated in human kidney tissue and experimental models, whereas C5b-9 promoted renal tubular epithelial pyroptosis in a trichloroethylene-associated experimental model [42,101]. Inflammasome activation, particularly in podocytes, has been implicated across several glomerular disease contexts [102]. Candidate therapeutic approaches include NLRP3 inhibition (e.g., MCC950), gasdermin D inhibition (e.g., disulfiram), P2X7 receptor antagonism, and downstream IL-1β or IL-18 blockade [103,104,105,106]. For example, disulfiram reduces ischemia–reperfusion AKI by suppressing caspase-11/GSDMD signaling [104]. In complement-driven settings, upstream complement inhibition may also reduce inflammasome activation and pyroptosis, although this remains context-dependent.

### 4.4. Ferroptosis

Ferroptosis is an iron-dependent form of regulated cell death driven by the accumulation of oxidized phospholipids and failure of cellular lipid peroxide detoxification (Figure 3) [6,107,108]. Labile ferrous iron (Fe+2) promotes radical-generating reactions. Hydroxyl radicals oxidize polyunsaturated fatty acids (PUFAs) incorporated in membrane phospholipids [109]. Acyl-CoA synthetase long-chain family 4 (ACSL4) and lysophosphatidylcholine acyltransferase 3 (LPCAT3) promote the incorporation of arachidonic and adrenic acids into phospholipids, particularly phosphatidylethanolamines, thereby increasing ferroptosis sensitivity [110,111,112]. Glutathione peroxidase 4 (GPX4), a selenium-containing glutathione peroxidase, suppresses ferroptosis by reducing phospholipid hydroperoxides to non-toxic lipid alcohols. Ferroptosis Suppressor Protein-1 (FSP1) regenerates reduced Coenzyme Q10 (CoQ10, ubiquinone), which acts as a parallel, glutathione-like radical-trapping antioxidant [113]. When GPX4 and FSP1 are overwhelmed or inhibited, lipid peroxides accumulate, causing membrane damage and cell death. Morphologically, ferroptotic cells display shrunken mitochondria, reduced cristae, and increased membrane density [5,6,114].

Renal pathology involves several ferroptosis-promoting stresses. Ischemia–reperfusion injury, cisplatin nephrotoxicity, and rhabdomyolysis-associated AKI are linked, in experimental evidence, to iron-dependent lipid peroxidation, ROS accumulation, and impairment of the GSH/GPX4 antioxidant system [108,115,116]. In rhabdomyolysis, myoglobin degradation in the kidney releases iron, promoting Fenton chemistry and lipid peroxidation in proximal tubular epithelial cells [116]. In diabetic kidney disease, hyperglycemia promotes renal tubular and glomerular cell ferroptosis through oxidative stress, iron overload, lipid peroxidation, and suppression of anti-ferroptotic pathways, including GPX4/Nrf2/SLC7A11 [117,118]. Complement may indirectly favor ferroptotic injury by promoting ROS generation and inflammatory amplification; in trichloroethylene-sensitized mice and C5b-9-treated human tubular epithelial cells, C5b-9 promoted tubular ferroptosis, at least partly through intracellular Ca^2+^ overload [119]. Conversely, ferroptosis generates oxidized phospholipids and lipid peroxidation products that may serve as inflammatory or oxidation-specific neoepitopes, although direct evidence that ferroptotic renal cells activate complement remains limited. Ferroptosis inhibitors, including ferrostatin-1, liproxstatin-1, and iron chelators such as deferoxamine, protect against experimental AKI and show emerging benefits in models of chronic kidney injury and fibrosis [108,116,118]. Selenomethionine has been shown experimentally to inhibit ferroptosis by supporting GPX4 biosynthesis and through GPX4-independent antioxidant effects; however, these findings are not kidney-specific and their relevance to renal ferroptosis remains to be established [120]. Combined targeting of ferroptosis and complement is therefore a plausible strategy for complement-amplified renal injury, but it should be framed as a hypothesis requiring experimental validation.

### 4.5. Autophagy

Autophagy is a conserved lysosome-dependent degradative process that maintains cellular homeostasis by removing damaged organelles and proteins. It is regulated by nutrient-sensing pathways such as the mammalian Target of Rapamycin (mTOR) and AMP-activated protein kinase (AMPK) pathways, as well as sirtuins (Figure 3) [121,122]. Under nutrient-rich conditions, mTOR suppresses autophagy [121,122]. Under cellular stress, mTOR inhibition and ULK1 complex activation initiate autophagosome formation [121,122,123]. Autophagosomes sequester damaged organelles and proteins and fuse with lysosomes, allowing degradation and recycling of their contents [124]. Importantly, autophagy is predominantly a cytoprotective process and should not itself be considered a form of regulated cell death; the term autophagy-dependent cell death should be restricted to settings in which the autophagic machinery is mechanistically required for cell death [5,125,126,127]. Autophagy crosstalk with the complement system: intracellular C3 can interact with ATG16L1 to regulate autophagy in pancreatic β cells, while C3-opsonized cytosolic bacteria can be recognized through C3–ATG16L1 interactions targeted for autophagy-dependent restriction [128,129,130,131]. These mechanisms, however, have been demonstrated predominantly in non-renal experimental systems.

In the kidney, autophagy maintains the integrity of podocytes and tubular cells [132,133,134]. Conditional deletion of autophagy genes leads to podocyte dysfunction, podocyte loss, proteinuria, and glomerulosclerosis, whereas upregulation of autophagy can protect against renal injury [132,133,134,135,136]. However, excessive or persistent autophagy can be deleterious [137,138]. In models of unilateral ureteral obstruction, prolonged tubular autophagy has been reported to contribute to tubular atrophy and interstitial fibrosis, although other studies suggest context-dependent anti-fibrotic effects [137,138,139]. Complement can modulate autophagy: C3a/C5a receptor signaling has been implicated in regulating mitophagy and macroautophagy, but the direction of the effect appears cell- and context-dependent. In experimental membranous nephropathy, sublytic MAC/C5b-9 can induce autophagosome formation in podocytes as a stress-response pathway but also impair lysosomal flux and convert this response into podocyte injury [34,129,140]. Experimental modulation of AMPK/mTOR-dependent autophagy with metformin and rapamycin has also attenuated podocyte injury in models of membranous nephropathy [140]. The interaction between autophagy and other death modalities is complex: autophagy may delay apoptosis and necroptosis by removing damaged organelles and regulating caspase- and RIPK-associated pathways, but excessive autophagy can contribute to autophagy-dependent cell death [125,126,127]. Pharmacologic modulation includes mTOR inhibitors such as rapamycin, AMPK activators such as metformin, and autophagy enhancers such as trehalose.

Morphological and Molecular Detection of Regulated Cell Death.

Each regulated cell death modality exhibits characteristic morphological and molecular features, but no single marker is generally sufficient for definitive identification. Reliable assessment therefore requires integration of morphology, biochemical markers, pathway-specific assays, and, where possible, functional perturbation (Table 2) [5,141]. Apoptosis is characterized by chromatin condensation, nuclear fragmentation, cell shrinkage, membrane blebbing, and the formation of apoptotic bodies [5,142]. TUNEL detects DNA fragmentation, annexin V binds externalized phosphatidylserine, and caspase activity assays, particularly detection of cleaved caspase-3, support activation of the apoptotic pathway [141,142]. However, these assays should be interpreted together because TUNEL and annexin V are not entirely specific for apoptosis and should be interpreted together with morphological and molecular evidence [141,142]. Transmission electron microscopy can show the classical ultrastructural features of apoptosis, including cell shrinkage, chromatin condensation, membrane blebbing, apoptotic bodies, and relatively preserved organelles [5,142].

Necroptosis is characterized by cellular swelling, organelle swelling or dilation, plasma membrane permeabilization or rupture, release of intracellular contents, and a relative absence of the classical apoptotic pattern of nuclear fragmentation [5,143]. Necroptosis involves activation of the Receptor-interacting serine-threonine kinase 1 (RIPK1)/Receptor-interacting serine-threonine kinase 3 (RIPK3)/Mixed Lineage Kinase Domain-Like pseudokinase (MLKL) pathway, particularly phosphorylation of RIPK3 and MLKL, oligomerization, and membrane translocation of MLKL. Immunohistochemistry or immunofluorescence can detect phospho-MLKL in experimental kidney injury and in human renal biopsy material, but phospho-MLKL should be considered evidence of activation of the necroptotic pathway rather than definitive proof of completed necroptotic cell death on its own [71,144,145]. Electron microscopy of necroptosis shows loss of plasma membrane integrity, organelle swelling, and cellular rupture rather than the compact nuclear fragmentation typical of apoptosis [5,143].

Pyroptosis is characterized by cell swelling, gasdermin-mediated plasma membrane pore formation, nuclear condensation, membrane rupture, and the release of inflammatory cytokines [88,146]. Cleaved gasdermin D (GSDMD-N) and activation of caspase-1 or caspase-4/5/11 are commonly used as markers of canonical or non-canonical pyroptosis, while caspase-3-mediated cleavage of gasdermin E marks secondary pyroptosis in GSDME-expressing cells [88,96,146]. IL-1β and IL-18 release can be quantified by ELISA, and imaging of ASC (Apoptosis-associated Speck-like protein containing a CARD) specks indicates inflammasome adaptor polymerization, although ASC specks reflect inflammasome activation rather than pyroptosis per se [88,147].

Ferroptosis lacks the nuclear fragmentation typical of apoptosis but shows dense, shrunken mitochondria with reduced or absent cristae [109,148]. Lipid peroxidation can be assessed by MDA (malondialdehyde), 4-HNE (4-hydroxynonenal), or C11-BODIPY fluorescence dyes, while GPX4 (glutathione peroxidase 4) expression/activity, labile iron, and glutathione levels are used to evaluate ferroptotic susceptibility [109,148,149]. Rescue by ferroptosis inhibitors such as ferrostatin-1 or liproxstatin-1 supports ferroptotic involvement but should be interpreted alongside lipid peroxidation, iron, GPX4/GSH, and morphological evidence rather than as a stand-alone confirmation [109,149].

**Table 2:** Candidate biopsy markers of complement activation and regulated cell death in renal disease.

Biological Process	Candidate Biopsy Marker/Readout	Suggested Method	Interpretation	Important Limitation
Classical pathway activation	C1q, C4d, IgG/IgM immune complex staining	Immunofluorescence or immunohistochemistry	Supports antibody- or immune complex-driven complement activation, especially in LN, anti-GBM disease, and some MN/IRGN cases	C1q/C4d alone do not prove pathogenic complement activation; pattern and disease context are essential
Lectin pathway activation	MBL, MASP2, ficolins, C4d	Immunohistochemistry, immunofluorescence, proteomics	Suggests lectin pathway contribution, relevant to IgAN and subsets of MN	Not routinely available in many renal pathology laboratories
Alternative pathway activation	C3, properdin, factor B/Bb, factor H/FHR proteins	Immunofluorescence, immunohistochemistry, mass spectrometry/proteomics	Supports amplification-loop activity, relevant to IgAN, AAV, IRGN, TMA/aHUS, and C3-dominant lesions	C3 deposition is common and not pathway-specific without additional markers
Terminal pathway activation	C5b-9/MAC	Immunohistochemistry or immunofluorescence	Indicates terminal complement activation and potential sublytic or lytic injury to podocytes, mesangial cells, endothelium, or tubules	MAC staining does not distinguish protective clearance, sublytic signaling, or completed cell lysis
Complement-regulator imbalance	CD46, CD55, CD59, factor H, factor I, CR1	Immunostaining, genetic testing, and complement functional assays	Reduced or abnormal regulation may support local susceptibility to complement-mediated injury	Tissue expression may be altered by chronic scarring and may not reflect inherited complement defects
Apoptosis	Cleaved caspase-3, cleaved caspase-8/9, TUNEL, annexin V, apoptotic bodies on EM	Immunohistochemistry, immunofluorescence, TUNEL, electron microscopy	Supports caspase-mediated programmed cell death; relevant to podocyte and tubular injury	TUNEL and annexin V are not fully specific; interpretation should combine morphology and pathway markers
Necroptosis	RIPK1/RIPK3 activation, phosphorylated MLKL, MLKL membrane localization	Immunohistochemistry, immunofluorescence, confocal microscopy	Suggests necroptosis-pathway activation in inflammatory renal injury	p-MLKL indicates pathway activation, but does not alone prove completed necroptotic death
Pyroptosis	NLRP3, ASC specks, cleaved caspase-1, caspase-4/5/11, cleaved gasdermin D, IL-1β, IL-18	Immunostaining, multiplex IF, RNA/protein assays	Supports inflammasome activation and inflammatory cell death; relevant to MN, IgAN, LN, AKI, and complement-amplified injury	ASC/NLRP3 may indicate inflammasome activation without definitive pyroptotic cell death
Ferroptosis	Reduced GPX4, reduced SLC7A11, ACSL4 upregulation, 4-HNE, MDA, lipid ROS, iron deposition	Immunohistochemistry, oxidative lipid staining, iron stains, proteomics	Suggests iron-dependent lipid-peroxidative injury, especially in tubular and podocyte stress states	Human renal-biopsy validation remains limited; oxidative stress is not specific for ferroptosis
Autophagy/impaired autophagic flux	LC3-II, p62/SQSTM1, Beclin-1, ATG proteins, autophagosomes on EM	Immunostaining, electron microscopy, transcript/protein analysis	May indicate adaptive or maladaptive autophagy in podocytes and tubular cells	LC3 or p62 accumulation may reflect either increased autophagy or blocked autophagic flux
Integrated complement–cell death signature	Co-localization of C5b-9/C3d with cleaved caspase-3, p-MLKL, GSDMD-N, 4-HNE, or LC3/p62	Multiplex immunofluorescence, spatial proteomics, digital pathology	May help define whether complement-positive lesions are associated with apoptotic, necroptotic, pyroptotic, ferroptotic, or autophagy-related injury	Promising mechanistic approach, but not yet standardized for routine diagnostic decision-making

Autophagy is identified by the formation of double-membrane autophagosomes. LC3-I is conjugated to phosphatidylethanolamine to form LC3-II, which associates with autophagosomal membranes; LC3-II turnover and p62/SQSTM1 degradation are widely used to assess autophagic flux [150,151,152]. Fluorescent reporters such as GFP-LC3 or tandem RFP-GFP-LC3 monitor autophagosome formation and flux, and electron microscopy can reveal autophagosomes containing cytoplasmic material or organelles [150,151,152]. However, caution is warranted because the accumulation of LC3-II or autophagosomes can reflect impaired lysosomal degradation or blocked autophagic flux rather than increased autophagy [150,151,152].

Multiple pathways may coexist within the same tissue or even the same cell population, and pathway transition may occur. Comprehensive analyses that combine morphology, biochemical markers, pathway-specific assays, and functional perturbation are therefore necessary to delineate cell death mechanisms [5,141]. Combining detection of complement deposition with cell death markers in renal biopsies may improve mechanistic interpretation of disease processes and help guide targeted therapy, although this remains an interpretive strategy rather than a fully standardized diagnostic approach [141,145].

## 5. Complement-Mediated Damage and Cell Death in Immune-Mediated Kidney Diseases

In immune-mediated kidney diseases, complement activation not only causes direct cytolysis but also orchestrates inflammatory and fibrotic responses [4,13,153,154]. The following sections focus on selected immune-mediated glomerular diseases in which complement activation intersects with regulated cell death pathways (Table 1); primary complement-mediated glomerulopathies, such as C3 glomerulopathy, are not reviewed in detail because their comprehensive pathophysiology, genetic basis, and complement-directed treatment fall outside the scope of this review.

### 5.1. Membranous Nephropathy (MN)

**Figure 4:**
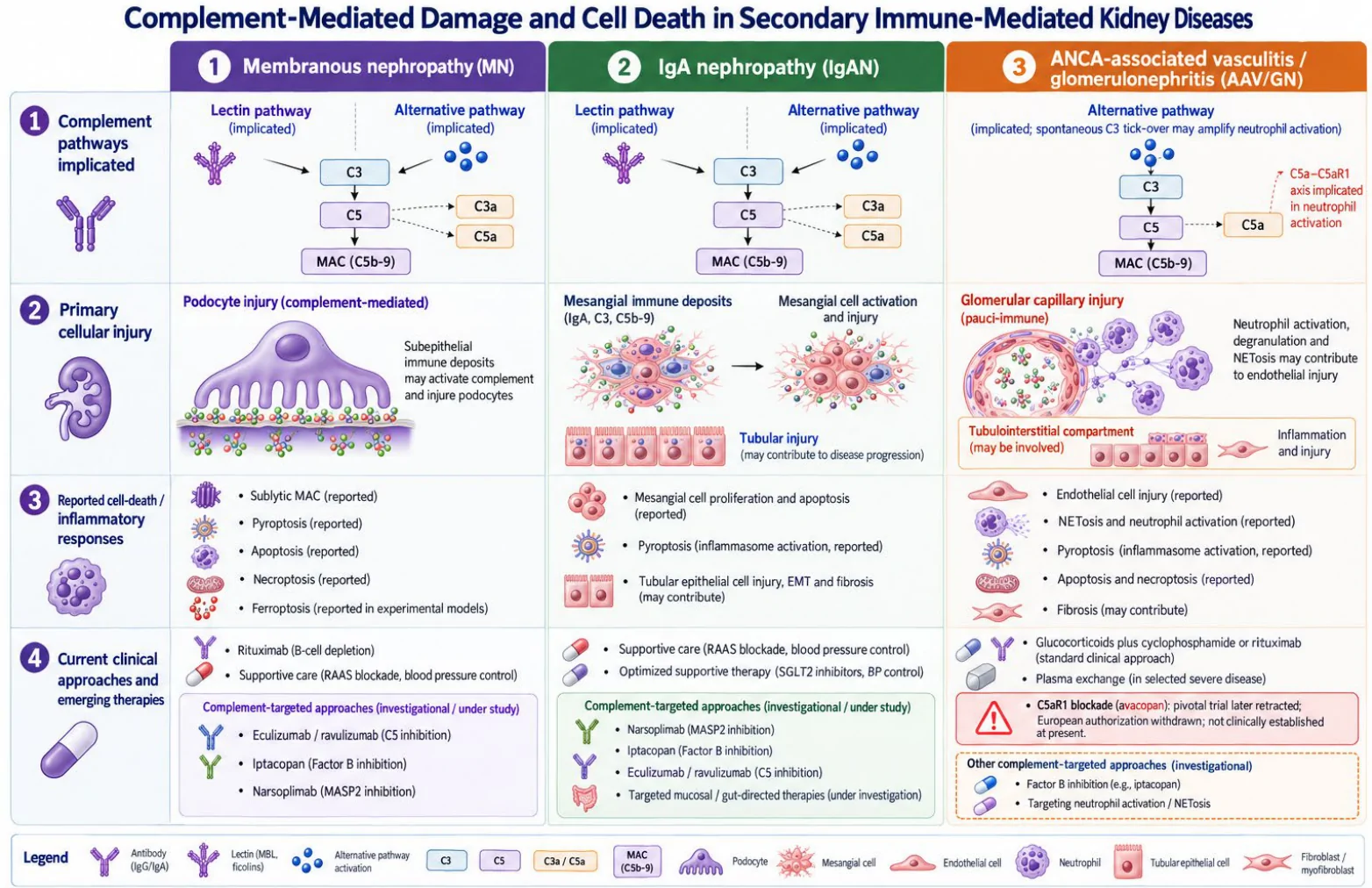
Complement-mediated damage and cell death in secondary immune-mediated kidney diseases. Membranous nephropathy, IgA nephropathy, and ANCA-associated vasculitis/glomerulonephritis. Schematic diagram only (AI-assisted illustration), no data-derived content. See Table 1, Table 2 and Table 3 and corresponding text sections for evidence details and references.

In MN, the complement system serves as a mediator linking autoantibody binding to podocyte injury (Figure 4). In PLA2R-associated MN, circulating autoantibodies bind PLA2R expressed on podocytes, resulting in subepithelial immune complex formation and local complement activation [155,156]. Interestingly, the pathway responsible for initiating complement activation has been long debated. Although anti-PLA2R antibodies are predominantly IgG4, a subclass with limited capacity to bind C1q and directly activate the classical pathway, this does not exclude classical pathway participation. IgG4 predominance is not necessarily exclusive; IgG subclass composition may vary among patients and during disease evolution, whereas evidence supports contributions from all three complement pathways [156,157]. Altered glycosylation of anti-PLA2R IgG4 can promote binding of mannose-binding lectin and activation of the lectin pathway, whereas factor B and properdin deposition and studies in patients with MBL deficiency support involvement of the alternative pathway [155,156]. More recent analyses of human MN biopsies have also demonstrated classical pathway activation, indicating that the relative contributions of the classical, lectin, and alternative pathways may vary among patients and disease stages [157]. Histologically, MN is characterized by uniform thickening of the glomerular basement membrane with a “spike and dome” appearance on a silver stain. Electron microscopy shows subepithelial electron-dense deposits and podocyte foot process effacement. Immunofluorescence demonstrates granular IgG4 and C3 [155,158]. C5b-9 deposition has also been demonstrated in glomeruli by complement-specific immunostaining and proteomic studies [155,156,157].

Podocyte response to complement is central to the pathogenesis. Experimental studies show that sublytic C5b-9 induces calcium-dependent signaling, cytoskeletal rearrangement, detachment, DNA damage, and shedding of podocyte membrane vesicles [13,34,36,37,42]. C3a- and C5a-induced podocyte pyroptosis has been demonstrated in human MN kidney tissue, cultured podocytes, and passive Heymann nephritis, with mitochondrial dysfunction, reactive oxygen species generation, and gasdermin-associated membrane injury implicated in this process [42]. Podocytes also undergo apoptosis secondary to complement-mediated mitochondrial injury [114]. Experimental evidence also suggests a role for ferroptosis in MN: passive Heymann nephritis is associated with increased renal iron and lipid peroxidation, reduced GPX4 expression, and other ferroptosis-associated changes, although the relevance of this pathway to human MN remains to be established [159,160]. However, evidence establishing necroptosis or ferroptosis as distinct pathogenic mechanisms in MN remains limited. Circulating C3a and glomerular podocyte C3aR expression are increased and correlate with disease severity. In vitro C3aR blockade attenuates MN plasma-induced podocyte injury, while C3aR antagonism reduces proteinuria and podocyte injury in experimental Heymann nephritis [161]. In advanced MN, complement activation contributes to tubulointerstitial inflammation and fibrosis via C5a-mediated activation of fibroblasts and pericytes [31,154,162].

Therapeutic approaches for MN have evolved from broad immunosuppression to targeted therapy. Rituximab depletes B cells that produce pathogenic autoantibodies; cyclophosphamide, combined with steroids, remains effective for patients at high risk of progressive kidney disease but carries toxicity [155,163]. Complement inhibitors are promising but remain investigational. Despite strong mechanistic evidence implicating complement in MN, no complement-directed therapy is currently established for routine treatment of primary MN, although many trials have paved the way towards that direction recently: eculizumab and ravulizumab blocking C5 cleavage; avacopan inhibiting C5aR1; iptacopan trial, inhibiting factor B; narsoplimab inhibiting MASP2.

### 5.2. IgA Nephropathy (IgAN)

IgAN follows a multi-hit hypothesis: (1) production of galactose-deficient IgA1 (Gd-IgA1) in the mucosa, (2) generation of autoantibodies against Gd-IgA1, (3) formation of circulating immune complexes, and (4) mesangial deposition leading to inflammation (Figure 4) [4,31,33,164]. Complement activation occurs at multiple steps, predominantly through the lectin and alternative pathways. Lectin pathway components MBL, MASP2, and ficolins bind aberrant O-glycans on IgA1, initiating complement activation. Alternative pathway activation amplifies complement deposition by stabilizing properdin. Factors regulating the alternative pathway, such as factor H and FHRs, determine the extent of activation [2,11,165].

Mesangial deposition of immune complexes triggers mesangial cell proliferation and matrix expansion. C5a and C3a promote recruitment of macrophages and neutrophils and upregulate adhesion molecules on glomerular endothelial cells. Experimental studies support a role for C3aR and C5aR1 signaling in mesangial activation. In cultured human mesangial cells, C3aR/C5aR blockade reduced IgA-induced proliferation and IL-6 and MCP-1 production, while genetic deficiency of these receptors in an experimental IgAN model reduced renal expression of several inflammatory and profibrotic mediators [164]. Sublytic membrane attack complex (MAC) deposition can induce mesangial cell apoptosis or proliferation, depending on MAC deposition density and expression of the complement regulatory protein CD59, as shown in animal models [57,58,166,167].

Podocytes exposed to IgA immune complexes and complement activation undergo foot process effacement and detachment. Tubular complement activation leads to tubular epithelial cell injury, contributing to the progression from glomerulonephritis to tubulointerstitial fibrosis. C5a triggers epithelial–mesenchymal transition-like changes, stimulates fibroblast proliferation and matrix production, and amplifies interstitial inflammation. In experimental IgA nephropathy, complement activation has also been associated with NLRP3 inflammasome activation, suggesting that complement links immune activation to inflammatory cell death and progressive renal injury [102,164]. Tubular complement activation may contribute to tubular epithelial injury and progression toward tubulointerstitial fibrosis. In experimental proteinuric nephropathy, C3a/C3aR signaling has been shown to promote epithelial-to-mesenchymal transition-like changes in tubular epithelial cells [168].

Proteinuria, hypertension, and impaired kidney function are well-established predictors of disease progression. Serum Gd-IgA1 is being investigated as a disease-specific biomarker, while complement activation markers, including serum C3a and sC5b-9 as well as urinary C5a, are being evaluated for their potential role in disease monitoring. In addition, genetic variants in complement pathway genes, including *CFH* and the *CFHR3–CFHR1* deletion, have been associated with disease susceptibility and clinical outcomes [4,33,165].

Treatment for IgAN includes optimization of blood pressure control, blockade of the renin–angiotensin–aldosterone system, and immunosuppression (corticosteroids, mycophenolate mofetil). Complement-directed therapy in IgA nephropathy is most advanced for the alternative and lectin pathways. Iptacopan, an oral factor B inhibitor, reduced proteinuria in the phase 3 APPLAUSE-IgAN trial and received accelerated approval for reduction of proteinuria in adults with primary IgAN at risk of rapid disease progression [169]. In contrast, although narsoplimab, an anti-MASP-2 antibody targeting the lectin pathway, reduced proteinuria in an earlier phase 2 IgAN study [170], the phase 3 ARTEMIS-IgAN trial was terminated after an interim analysis failed to meet its prespecified primary endpoint. Terminal pathway inhibition with ravulizumab, an anti-C5 antibody, reduced proteinuria in a phase 2 randomized trial [171], and positive proteinuria results have subsequently been reported from the prespecified interim analysis of the ongoing phase 3 I CAN trial, while the final kidney function outcome remains pending [172]. Other factor B–directed approaches, such as sefaxersen/IONIS-FB-LRx, an antisense inhibitor of complement factor B, have also demonstrated proteinuria reduction in a phase 2 study and are undergoing phase 3 evaluation [173]. Finally, targeted-release budesonide and anti-APRIL therapies may indirectly attenuate complement activation by reducing mucosal Gd-IgA1 production and immune complex formation, but they are not direct complement inhibitors [4].

### 5.3. ANCA-Associated Vasculitis/Glomerulonephritis (AAV)

AAV is driven by autoantibodies directed against neutrophil cytoplasmic enzymes, primarily proteinase 3 (PR3) and myeloperoxidase (MPO) [174,175,176,177]. ANCA-mediated activation of primed neutrophils promotes degranulation and the release of neutrophil extracellular traps (NETs), which expose autoantigens and promote complement activation (Figure 4). NETs provide a scaffold for alternative pathway activation through properdin, while NET components, particularly histones, can directly cleave C3 [178,179,180,181,182]. C5a is a central amplifier of this inflammatory cascade, priming neutrophils by increasing surface expression of ANCA antigens and enhancing their adhesion, activation, and degranulation. Signaling through C5aR1 on neutrophils and monocytes further promotes oxidative burst, cytokine production, and tissue factor expression, thereby linking inflammation to thrombosis, while establishing a positive-feedback loop between neutrophil activation and the alternative complement pathway [11,176,183,184,185].

Within the renal vasculature, ANCA-activated neutrophils adhere to the endothelium and transmigrate into the vessel wall, releasing lytic enzymes and ROS that injure endothelial cells and produce the characteristic pauci-immune necrotizing and crescentic glomerulonephritis. Complement activation results in the deposition of C3, C5b-9, and properdin along glomerular capillary loops and small-vessel walls, thereby further amplifying vascular injury. Endothelial cells exposed to sublytic MAC release von Willebrand factor and express adhesion molecules, promoting additional neutrophil recruitment and sustaining the inflammatory response [162,186].

Regulated cell death may participate directly in this complement–neutrophil amplification loop. Experimental studies have shown that ANCA-induced NET formation can depend on RIPK1-, RIPK3-, and MLKL-mediated necroptotic signaling. In murine AAV models, disruption of this pathway reduced NET formation, alternative pathway complement activation, endothelial injury, and vasculitis, while corresponding evidence of pathway activation was also demonstrated in human AAV kidney biopsies [75]. Thus, among regulated cell death pathways, necroptosis currently has particularly strong mechanistic support in AAV, linking ANCA-induced neutrophil activation to NET generation, complement amplification, and endothelial injury.

Clinically, AAV manifests with rapidly progressive glomerulonephritis, hematuria, proteinuria, and extrarenal features such as pulmonary hemorrhage and neuropathy. Disease activity correlates with plasma levels of Bb, C3a, C5a, and soluble C5b-9 [187,188,189,190]. Reduced factor H levels and MPO-mediated oxidative impairment of factor H contribute to the uncontrolled activation of the alternative complement pathway [187,188,189,190,191,192,193].

Standard treatment consists of high-dose corticosteroids combined with cyclophosphamide or rituximab, with plasma exchange reserved for selected patients with severe disease. Complement-directed therapy in AAV has focused particularly on the C5a–C5aR1 axis. Avacopan, an oral C5aR1 antagonist, was initially supported by the ADVOCATE trial as a glucocorticoid-sparing adjunct. However, the ADVOCATE publication was retracted in June 2026 after undisclosed post-unblinding readjudication of primary endpoint assessments was identified, and the pivotal trial data are therefore no longer considered reliable evidence of efficacy. Importantly, this retraction concerned trial conduct and data integrity, not the emergence of a new safety signal. Separately, following an Article 20 review, the European Medicines Agency concluded that avacopan’s clinical benefit could no longer be reliably demonstrated and that its benefits were therefore no longer proven to outweigh its risks; the European Commission consequently revoked its EU marketing authorization on 4 August 2026. The regulatory assessment also considered recognized safety concerns, particularly drug-induced liver injury and vanishing bile duct syndrome, which require distinction from the efficacy-related data integrity concerns that prompted reassessment of the pivotal evidence [194]. Other complement inhibitors under investigation include iptacopan and eculizumab [195]. Because neutrophil activation is central, therapies targeting NET formation, neutrophil elastase, and peptidylarginine deiminase (PAD) may also indirectly reduce complement activation [196].

### 5.4. Lupus Nephritis (LN)

**Figure 5:**
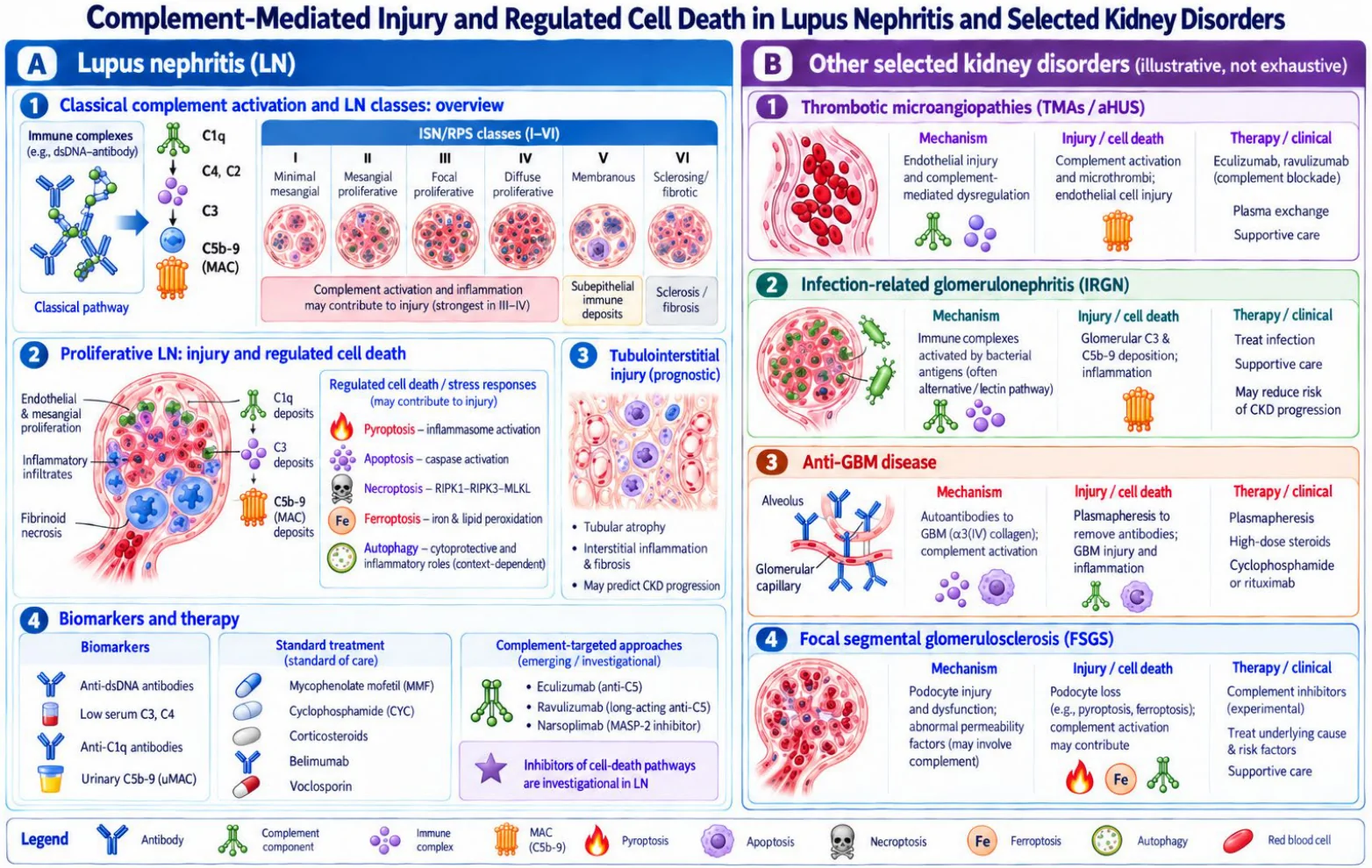
Complement-mediated injury and regulated cell death in lupus nephritis, thrombotic microangiopathies, membranoproliferative glomerulonephritis, anti-GBM disease, and focal segmental glomerulosclerosis. Abbreviations: LN, lupus nephritis/ISN/RPS, International Society of Nephrology/Renal Pathology Society, aHUS, atypical hemolytic uremic syndrome, MAC, membrane attack complex; CKD, chronic kidney disease; GBM, glomerular basement membrane. Schematic diagram only (AI-assisted illustration), no data-derived content. See Table 1, Table 2 and Table 3 and corresponding text sections for evidence details and references.

LN results from deposition of diverse immune complexes in the glomeruli and the activation of complement via the classical pathway (Figure 5). The International Society of Nephrology/Renal Pathology Society (ISN/RPS) classifies LN into six classes based on histology, ranging from minimal mesangial (class I) to advanced sclerosing (class VI). Each class is associated with distinct patterns of complement deposition and cell death [197,198,199,200].

In proliferative classes (III and IV), glomerular capillaries are occluded by proliferating endothelial and mesangial cells, inflammatory infiltrates, and fibrinoid necrosis. Complement activation with glomerular deposition of C1q, C3, and C5b-9 is a hallmark of proliferative LN, demonstrated as part of the “full-house” pattern in immunofluorescence. At sublytic concentrations, C5b-9 (MAC) can activate endothelial cells, inducing chemokine production and adhesion molecule expression. In primed human endothelial cells, internalized MAC can also trigger NLRP3 inflammasome assembly, caspase-1 activation, gasdermin-D processing, and IL-1β release [14]. C5b-9 can also be detected in glomeruli, although glomerular C5b-9 deposition does not necessarily reflect ongoing terminal pathway activation [201]. Human studies additionally demonstrate alternative pathway activation in LN [202,203].

Impaired clearance of apoptotic bodies exposes nuclear antigens, perpetuating autoimmunity and complement activation. Necroptosis can also be induced in endothelial and epithelial cells by interferon-α, TNF-α, and complement activation. Emerging evidence further implicates ferroptosis, supported by iron overload, lipid peroxidation, and reduced GPX4 activity, resulting in renal tubular epithelial injury (Figure 5). Autophagy appears protective during early disease but becomes maladaptive as chronic inflammation progresses [5,6,197,200,204]. Pyroptosis-related signaling has also been demonstrated in LN: GSDMD activation has been observed in neutrophils within human LN kidney biopsies and in experimental lupus, with oxidized mitochondrial DNA implicated mechanistically in gasdermin activation [205].

Class V (membranous) LN is characterized by subepithelial immune complex deposition and complement activation, resembling those observed in primary membranous nephropathy. In contrast, Class VI represents advanced chronic kidney injury, with extensive glomerulosclerosis, interstitial fibrosis, and minimal residual inflammatory activity. Beyond glomerular lesions, tubulointerstitial injury is a major determinant of long-term renal outcome, with local complement activation promoting tubular atrophy, interstitial inflammation, and fibrosis [198,199,206].

Serological markers such as anti-double-stranded DNA (dsDNA) antibodies, complement levels (C3, C4), anti-C1q antibodies, and urinary complement components (C5b-9) aid in disease monitoring. Low serum complement levels reflect consumption and correlate with activity [199].

Treatment of LN includes immunosuppression (mycophenolate mofetil, cyclophosphamide, corticosteroids), belimumab (anti-BAFF), and voclosporin (a calcineurin inhibitor). Complement-targeting therapies are being explored: eculizumab has been used off-label; ravulizumab and obinutuzumab (anti-CD20) are under study; narsoplimab may reduce complement activation by inhibiting MASP2 [207]. Addressing cell death pathways via inhibitors of necroptosis, pyroptosis, and ferroptosis may complement immunosuppression [4,199].

### 5.5. Interplay Among Death Pathways and Complement

**Figure 6:**
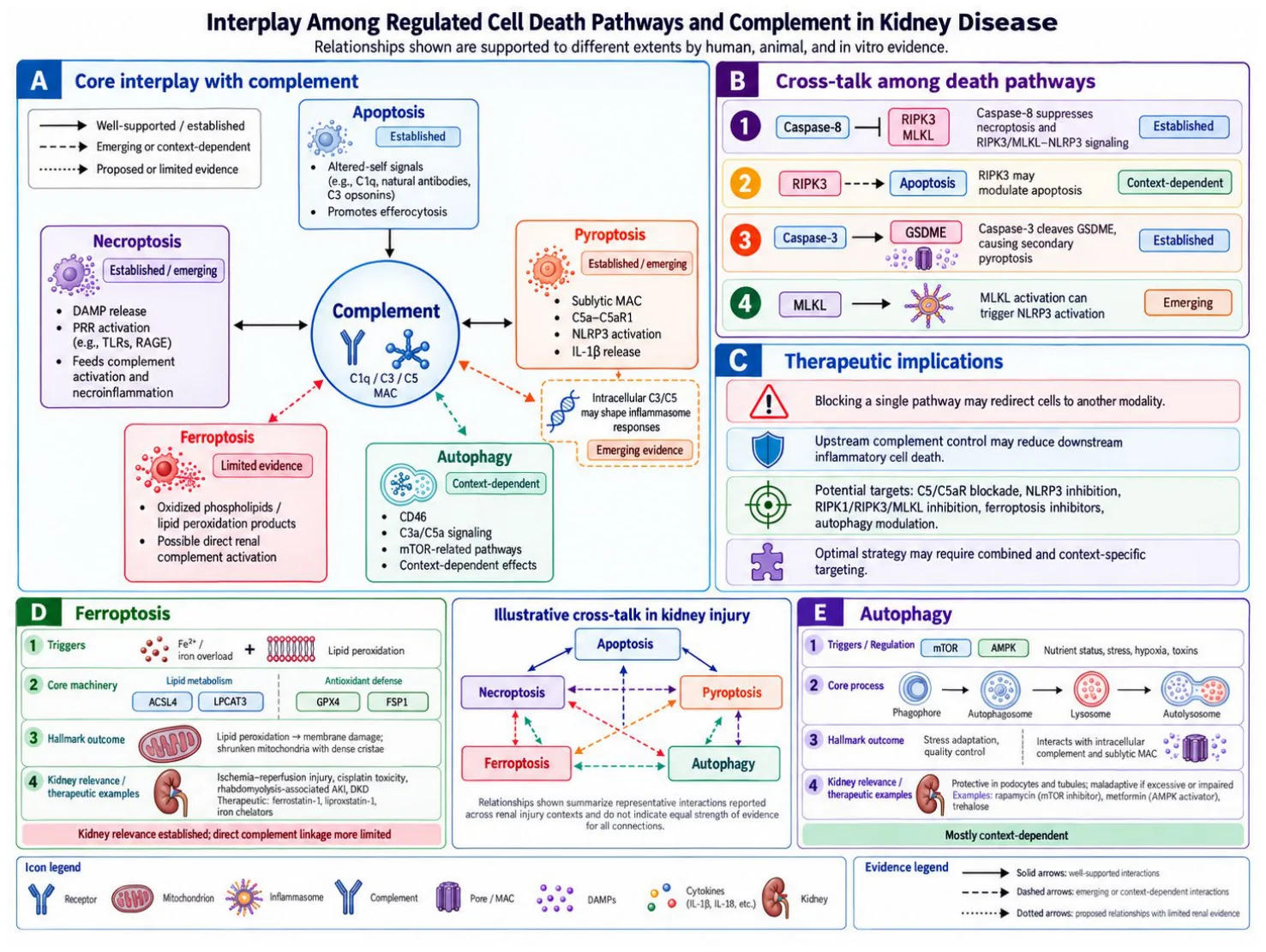
Interplay among regulated cell death pathways and complement in kidney disease. (**A**) Core interactions between complement activation and regulated cell death pathways, including apoptosis, necroptosis, pyroptosis, ferroptosis, and autophagy. (**B**) Crosstalk among regulated cell death pathways, highlighting representative interactions involving caspase-8, RIPK3, caspase-3/GSDME, and MLKL/NLRP3 signaling. (**C**) Therapeutic implications of complement–cell death crosstalk and potential targets for combined or context-specific intervention. (**D**) Ferroptosis mechanisms and renal relevance. (**E**) Autophagy regulation, cellular effects, and renal relevance. Solid arrows indicate relatively well-supported interactions; dashed arrows indicate emerging or context-dependent relationships; dotted arrows indicate proposed relationships supported by limited renal evidence. The schematic integrates evidence from human, animal, and in vitro studies and does not imply equivalent strength of evidence for all depicted interactions.

The interplay between complement and regulated cell death forms a network of positive and negative feedback loops (Figure 6). Apoptotic cells expose altered-self signals that are recognized by natural antibodies, C1q, C3 fragments, and other complement-related opsonins, thereby promoting efficient efferocytosis, namely the non-inflammatory phagocytic clearance of apoptotic cells. Failure of this clearance process allows apoptotic cells to progress to late apoptosis or secondary necrosis, resulting in the release of intracellular danger signals, enhanced complement activation, and amplification of inflammation [10,129]. Necroptosis and pyroptosis release Damage-Associated Molecular Patterns (DAMPs) that activate pattern-recognition receptors, while necrotic debris can activate complement through multiple pathways, creating a self-amplifying cycle of necroinflammation [70,129]. Complement can also promote inflammatory cell death signaling: sublytic MAC and C5a/C5aR1 signaling can activate the NLRP3 inflammasome and trigger IL-1β release, and intracellular complement pools, including C3 and C5, may shape inflammasome responses [208]. Ferroptosis generates oxidized phospholipids and lipid peroxidation products that may act as inflammatory or oxidation-specific neoepitopes, but direct evidence that ferroptotic renal cells activate complement or favor MAC insertion remains limited [209,210]. Autophagy intersects with complement regulation through molecules such as CD46, which functions both as a complement regulator and as an autophagy-inducing receptor. CD46 internalization, downregulation, or degradation may reduce complement protection in selected contexts. Conversely, complement fragments and receptors can modulate autophagy and cellular metabolism through C3a/C5a receptor signaling and mTOR-related pathways, with effects that vary by cell type and disease context [129,211].

Crosstalk also occurs between death pathways themselves: caspase-8 suppresses necroptosis and inflammatory RIPK3/MLKL–NLRP3 signaling [212,213]; RIPK3 may regulate apoptosis [214]; caspase-3 can cleave gasdermin E to induce pyroptosis, converting an apoptotic signal [96]; MLKL activation can trigger NLRP3 activation [215]. These interactions provide potential therapeutic targets: inhibiting a single pathway may shift cell death to another modality unless upstream triggers, such as complement activation, are also controlled [70,212].

## 6. Conclusions

This review underscores several key themes. First, complement is not a monolithic lytic cascade but a multifaceted system with roles in homeostasis, immunity, inflammation, and tissue repair. Its activation occurs through three pathways that integrate signals from antibodies, lectins, and spontaneous tick-over. Effector mechanisms include opsonization, anaphylatoxin-mediated inflammation, and MAC formation, all of which influence kidney physiology and pathophysiology. Intracellular complement, the complosome, adds an additional layer of regulation.

Second, regulated cell death pathways—apoptosis, necroptosis, pyroptosis, ferroptosis, and autophagy—are intricately linked to complement. Complement effectors can trigger or modulate cell death, and cell death releases signals that activate complement. The balance among these pathways determines the outcome of renal injury and fibrosis. Recognizing the interplay and crosstalk provides opportunities for therapeutic intervention.

Third, complement dysregulation underpins a spectrum of kidney diseases from immune complex–mediated glomerulonephritides (membranous nephropathy, IgA nephropathy, lupus nephritis) to pauci-immune vasculitides (ANCA) and thrombotic microangiopathies.

Finally, future research must integrate complement biology and cell death mechanisms to achieve precision nephrology. By embracing this complexity, clinicians and researchers can develop targeted therapies that preserve renal function and improve patient outcomes, moving to a nuanced modulation of complement and cell death (Table 3).

**Table 3:** Therapeutic targets linking complement and regulated cell death in renal disease.

Target	Drug/Class	Mechanistic Relevance	Disease Relevance	Representative Quantitative Clinical Effect	Current Evidence/Status
C5	Eculizumab; ravulizumab	Prevents C5 cleavage, reducing C5a generation and C5b-9/MAC formation	Complement-mediated TMA/aHUS; investigated in IgAN and other complement-amplified GN	In prospective eculizumab aHUS studies, 80% of patients in one cohort achieved TMA event-free status at week 26; in the progressive-TMA cohort, mean eGFR increased by 32 mL/min/1.73 m^2^ at week 26 [216]. In adult complement-inhibitor-naïve aHUS, ravulizumab produced complete TMA response in 53.6% (30/56; 95% CI 39.6–67.5) by week 26 [217]. In phase 2 IgAN, ravulizumab produced a 30.1% relative reduction in proteinuria versus placebo at week 26 (90% CI 13.7–43.5) [171].	Established for complement-mediated TMA/aHUS; investigational/emerging in glomerular diseases such as IgAN
C5a receptor 1 (C5aR1)	Avacopan	Inhibits C5a-mediated neutrophil activation and inflammatory amplification	AAV/GN	ADVOCATE originally reported remission at week 26 in 72.3% vs 70.1% and sustained remission at week 52 in 65.7% vs 54.9% with avacopan versus prednisone taper. However, the pivotal publication was subsequently retracted. EMA concluded that the pivotal data could no longer reliably demonstrate efficacy, and the EU marketing authorization was revoked on 4 August 2026. (European Medicines Agency (EMA)).	Previously authorized in the EU; efficacy evidence now uncertain because the pivotal trial is no longer considered reliable in the EU
Factor B	Iptacopan; factor-B inhibitors	Selective inhibition of alternative pathway amplification	IgAN; other alternative pathway-amplified disease	In APPLAUSE-IgAN, iptacopan reduced 24 h UPCR by 38.3% versus placebo at month 9 (95% CI 26.0–48.6; *p* < 0.001) [169]. At 24 months, annual eGFR slope was −3.10 vs −6.12 mL/min/1.73 m^2^/year, difference 3.02 (95% CI 2.02–4.01); the composite kidney-failure endpoint occurred in 21.4% vs 33.5%, HR 0.57 (95% CI 0.40–0.81) [218].	Phase 3 clinical evidence in IgAN
MASP-2	Narsoplimab	Lectin pathway inhibition	IgAN; other lectin pathway-positive renal disease	In phase 3 ARTEMIS-IgAN, week-36 24 h UPE changed by −21.3% with narsoplimab vs −16.6% with placebo; between-group difference −5.6 percentage points (95% CI −21.6 to 13.8; *p* = 0.5447). The primary endpoint was therefore not met and the trial was terminated.	Negative phase 3 efficacy result in IgAN; investigational elsewhere
C1s/classical pathway	Sutimlimab and other C1s inhibitors	Classical pathway inhibition	Potential relevance to antibody/immune complex nephropathies	No established quantitative renal clinical efficacy estimate in the glomerular diseases considered in this review.	Investigational
C3/proximal complement activation	C3 inhibitors and proximal complement blockers	Blocks convergence of the three complement pathways	Potentially relevant to complement-amplified GN	No established quantitative efficacy estimate within the selected disease scope of this review.	Disease-specific clinical development; not established across the diseases reviewed
B cells/autoantibody production	Rituximab; obinutuzumab	Reduces pathogenic autoantibody production upstream of complement activation	MN, AAV, LN	In MENTOR, complete/partial MN remission at 24 months occurred in 60% with rituximab vs 20% with cyclosporine; risk difference 40 percentage points (95% CI 25–55) [163]. In REGENCY LN, complete renal response at week 76 was 46.4% with obinutuzumab vs 33.1% with placebo, adjusted difference 13.4 percentage points (95% CI 2.0–24.8) [219].	Strong disease-specific clinical evidence; effect on complement is indirect
BAFF	Belimumab	Reduces B-cell survival and autoantibody activity	Lupus nephritis	BLISS-LN: primary efficacy renal response at week 104 43% vs 32% with placebo (OR 1.6, 95% CI 1.0–2.3); complete renal response 30% vs 20% (OR 1.7, 95% CI 1.1–2.7) [220].	Established adjunctive therapy in LN; indirect complement effect
Calcineurin pathway/podocyte stabilization	Voclosporin	Immunomodulation and podocyte stabilization	Lupus nephritis	AURORA-1: complete renal response at week 52 occurred in 41% with voclosporin vs 23% with placebo, OR 2.65 (95% CI 1.64–4.27; *p* < 0.0001) [221].	Established in LN; not a direct complement or regulated cell death inhibitor
Pathogenic IgG	Imlifidase	Cleaves IgG and rapidly decreases pathogenic autoantibody burden upstream of complement activation	Anti-GBM disease; antibody-mediated transplantation settings	In a phase 2a single-arm anti-GBM study, 10/15 patients (67%) were dialysis independent at 6 months versus 18% in a historical control cohort (*p* < 0.001) [222].	Promising but nonrandomized evidence; requires controlled confirmation
NLRP3 inflammasome	MCC950-like inhibitors	Inhibition of inflammasome activation/pyroptotic signaling	Experimental MN, IgAN, LN, AKI	No renal clinical trial efficacy estimate.	Preclinical/experimental
Gasdermin D	Disulfiram-like or gasdermin-targeted approaches	Inhibition of pyroptotic pore formation	Inflammatory renal injury	No renal clinical trial efficacy estimate demonstrating benefit through GSDMD inhibition.	Preclinical/experimental
IL-1β/IL-18 axis	IL-1 or IL-18 pathway blockade	Downstream inflammasome cytokine inhibition	Pyroptosis-associated renal inflammation	No established quantitative clinical efficacy in the glomerular diseases reviewed.	Limited/extrapolated clinical evidence
RIPK1/RIPK3/MLKL	RIPK1, RIPK3 and MLKL inhibitors	Necroptosis inhibition	AKI, vasculitis, LN and other necroinflammatory renal injury	No renal clinical trial efficacy estimate.	Preclinical/experimental
Ferroptosis machinery	Ferrostatin-1, liproxstatin-1, iron chelation, GPX4/Nrf2/SLC7A11-supporting strategies	Ferroptosis inhibition	Experimental AKI, DKD, LN and podocyte injury	No clinical trial has established renal benefit specifically through ferroptosis inhibition.	Preclinical/experimental
Autophagy pathways	mTOR/AMPK-directed or other autophagy-modulating approaches	Modulation of adaptive/maladaptive autophagy	Podocyte and tubular stress	No renal clinical trial establishes efficacy specifically through modulation of autophagic flux in these diseases.	Experimental/indirect clinical relevance
Combined complement + regulated cell death targeting	Rational combination strategies	Simultaneous inhibition of upstream complement activation and downstream regulated cell death	Severe complement-amplified renal injury	No clinical efficacy data.	Hypothesis-generating

## Data Availability

No new data were created or analyzed in this study. Data sharing is not applicable to this article.

## References

[1] Mathern D.R., Heeger P.S. (2015). Molecules Great and Small: The Complement System. Clin. J. Am. Soc. Nephrol. CJASN.

[2] Merle N.S., Church S.E., Fremeaux-Bacchi V., Roumenina L.T. (2015). Complement System Part I-Molecular Mechanisms of Activation and Regulation. Front. Immunol..

[3] Kolev M., Friec G.L., Kemper C. (2014). Complement—tapping into new sites and effector systems. Nat. Rev. Immunol..

[4] Vivarelli M., Barratt J., Beck L.H., Fakhouri F., Gale D.P., de Jorge E.G., Mosca M., Noris M., Pickering M.C., Susztak K. (2024). The role of complement in kidney disease: Conclusions from a Kidney Disease: Improving Global Outcomes (KDIGO) Controversies Conference. Kidney Int..

[5] Galluzzi L., Vitale I., Aaronson S.A., Abrams J.M., Adam D., Agostinis P., Alnemri E.S., Altucci L., Amelio I., Andrews D.W. (2018). Molecular mechanisms of cell death: Recommendations of the Nomenclature Committee on Cell Death 2018. Cell Death Differ..

[6] Long Y., Tan G., Li X., Xiang B., Tao Y., Xie Z., Zhang X. (2024). Programmed cell death: Molecular mechanisms, biological functions, diseases, and therapeutic targets. MedComm.

[7] Berger S.P., Daha M.R. (2007). Complement in glomerular injury. Semin. Immunopathol..

[8] Borza D.B. (2017). Glomerular basement membrane heparan sulfate in health and disease: A regulator of local complement activation. Matrix Biol..

[9] Dunkelberger J.R., Song W.C. (2010). Complement and its role in innate and adaptive immune responses. Cell Res..

[10] Botto M., Dell’Agnola C., Bygrave A.E., Thompson E.M., Cook H.T., Petry F., Loos M., Pandolfi P.P., Walport M.J. (1998). Homozygous C1q deficiency causes glomerulonephritis associated with multiple apoptotic bodies. Nat. Genet..

[11] Roumenina L., Bigot S., Brachemi S., Frémeaux-Bacchi V., Lesavre P., Halbwachs M. (2011). Complement alternative pathway acts as a positive feedback amplification of neutrophil activation. Blood.

[12] Stenson E.K., Kendrick J., Dixon B., Thurman J.M. (2023). The complement system in pediatric acute kidney injury. Pediatr. Nephrol..

[13] Xie C.B., Jane-Wit D., Pober J.S. (2020). Complement Membrane Attack Complex: New Roles, Mechanisms of Action, and Therapeutic Targets. Am. J. Pathol..

[14] Bp K., Hughes T.R., Triantafilou M., Morgan B.P. (2013). The complement membrane attack complex triggers intracellular Ca2+ fluxes leading to NLRP3 inflammasome activation. J. Cell Sci..

[15] Morgan B.P. (1989). Complement membrane attack on nucleated cells: Resistance, recovery and non-lethal effects. Biochem. J..

[16] Verma J., Arora V., Marwaha V., Kumar A., Das N. (2005). Association of leukocyte CR1 gene transcription with the disease severity and renal involvement in systemic lupus erythematosus. Lupus.

[17] Guthridge J.M., Rakstang J.K., Young K.A., Hinshelwood J., Aslam M., Robertson A., Gipson M.G., Sarrias M.R., Moore W.T., Meagher M. (2001). Structural studies in solution of the recombinant N-terminal pair of short consensus/complement repeat domains of complement receptor type 2 (CR2/CD21) and interactions with its ligand C3dg. Biochemistry.

[18] Liu D., Zhu J.Y., Niu Z.X. (2008). Molecular structure and expression of anthropic, ovine, and murine forms of complement receptor type 2. Clin. Vaccine Immunol..

[19] Lukacsi S., Gerecsei T., Balazs K., Francz B., Szabo B., Erdei A., Bajtay Z. (2020). The differential role of CR3 (CD11b/CD18) and CR4 (CD11c/CD18) in the adherence, migration and podosome formation of human macrophages and dendritic cells under inflammatory conditions. PLoS ONE.

[20] Calderwood J.W., Williams J.M., Morgan M.D., Nash G.B., Savage C.O. (2005). ANCA induces beta2 integrin and CXC chemokine-dependent neutrophil-endothelial cell interactions that mimic those of highly cytokine-activated endothelium. J. Leukoc. Biol..

[21] van Lookeren Campagne M., Wiesmann C., Brown E.J. (2007). Macrophage complement receptors and pathogen clearance. Cell Microbiol..

[22] Tenner A.J., Johswich K.O., Ager R.R., Reis E.S., Köhl J. (2009). The role of the anaphylatoxins in health and disease. Mol. Immunol..

[23] Bajic G., Degn S.E., Thiel S., Andersen G.R. (2015). Complement activation, regulation, and molecular basis for complement-related diseases. EMBO J..

[24] Armento A., Honisch S., Panagiotakopoulou V., Sonntag I., Jacob A., Bolz S., Kilger E., Deleidi M., Clark S., Ueffing M. (2020). Loss of Complement Factor H impairs antioxidant capacity and energy metabolism of human RPE cells. Sci. Rep..

[25] Hannan J.P., Laskowski J., Thurman J.M., Hageman G.S., Holers V.M. (2016). Mapping the Complement Factor H-Related Protein 1 (CFHR1):C3b/C3d Interactions. PLoS ONE.

[26] Medjeral-Thomas N., Pickering M.C. (2016). The complement factor H-related proteins. Immunol. Rev..

[27] Brodszki N., Frazer-Abel A., Grumach A.S., Kirschfink M., Litzman J., Perez E., Seppanen M.R.J., Sullivan K.E., Jolles S. (2020). European Society for Immunodeficiencies (ESID) and European Reference Network on Rare Primary Immunodeficiency, Autoinflammatory and Autoimmune Diseases (ERN RITA) Complement Guideline: Deficiencies, Diagnosis, and Management. J. Clin. Immunol..

[28] Kolev M., Dimeloe S., Le Friec G., Navarini A., Arbore G., Povoleri G.A., Fischer M., Belle R., Loeliger J., Develioglu L. (2015). Complement Regulates Nutrient Influx and Metabolic Reprogramming during Th1 Cell Responses. Immunity.

[29] Jalink M., de Boer E.C.W., Evers D., Havinga M.Q., Vos J.M.I., Zeerleder S., de Haas M., Jongerius I. (2021). Halting targeted and collateral damage to red blood cells by the complement system. Semin. Immunopathol..

[30] Qin X., Goldfine A., Krumrei N., Grubissich L., Acosta J., Chorev M., Hays A.P., Halperin J.A. (2004). Glycation inactivation of the complement regulatory protein CD59: A possible role in the pathogenesis of the vascular complications of human diabetes. Diabetes.

[31] Gao S., Cui Z., Zhao M.H. (2020). The Complement C3a and C3a Receptor Pathway in Kidney Diseases. Front. Immunol..

[32] Xu B., Zhou Z., Xiao Y., Liu Q., Xiao T., Lv Z., Wang H. (2026). The Pleiotropic Effect of Complement C5a-C5aR1 Pathway in Diseases: From Immune Regulation to Targeted Therapy. Int. J. Mol. Sci..

[33] Liu L., Zhang Y., Duan X., Peng Q., Liu Q., Zhou Y., Quan S., Xing G. (2014). C3a, C5a renal expression and their receptors are correlated to severity of IgA nephropathy. J. Clin. Immunol..

[34] Hf J., Li Z.H., Chen X.C., Zhao X.L., Zhong Z., Yang C., Wu H.L., An N., Li W.Y., Liu H.-F. (2017). Blockage of the lysosome-dependent autophagic pathway contributes to complement membrane attack complex-induced podocyte injury in idiopathic membranous nephropathy. Sci. Rep..

[35] Soane L., Cho H.J., Niculescu F., Rus H., Shin M.L. (2001). C5b-9 terminal complement complex protects oligodendrocytes from death by regulating Bad through phosphatidylinositol 3-kinase/Akt pathway. J. Immunol..

[36] Sj W., Durvasula R., Petermann A., Hiromura K., Couser W.G., Shankland S.J. (2003). DNA damage is a novel response to sublytic complement C5b-9-induced injury in podocytes. J. Clin. Investig..

[37] Reiser J., Singh G., Altintas M.M., Yu H., Fernandez I.C., Gu C., Wilson C., Srivastava S.K., Dietrich A., Walz K. (2013). Transient receptor potential channel 6 (TRPC6) protects podocytes during complement-mediated glomerular disease. J. Biol. Chem..

[38] Li Y., Fang Y., Liu J. (2023). Downregulation of TRPC6 regulates ERK1/2 to prevent sublytic C5b-9 complement complex-induced podocyte injury through activating autophagy. Exp. Ther. Med..

[39] Wieder N., Greka A. (2016). Calcium, TRPC channels, and regulation of the actin cytoskeleton in podocytes: Towards a future of targeted therapies. Pediatr. Nephrol..

[40] Fosbrink M., Cudrici C., Tegla C.A., Soloviova K., Ito T., Vlaicu S., Rus V., Niculescu F., Rus H. (2009). Response gene to complement 32 is required for C5b-9 induced cell cycle activation in endothelial cells. Exp. Mol. Pathol..

[41] Hughes J., Nangaku M., Alpers C.E., Shankland S.J., Couser W.G., Johnson R.J. (2000). C5b-9 membrane attack complex mediates endothelial cell apoptosis in experimental glomerulonephritis. Am. J. Physiol. Ren. Physiol..

[42] Liu Z., Li L., Lv D., Jiang S., Hou Q., Zhang L., Li S., Zhu X., Xu X., Wen J. (2022). Complement induces podocyte pyroptosis in membranous nephropathy by mediating mitochondrial dysfunction. Cell Death Dis..

[43] Adler S., Baker P.J., Johnson R.J., Ochi R.F., Pritzl P., Couser W.G. (1986). Complement membrane attack complex stimulates production of reactive oxygen metabolites by cultured rat mesangial cells. J. Clin. Investig..

[44] Mühlig A.K., Keir L.S., Abt J.C., Heidelbach H.S., Horton R., Welsh G.I., Meyer-Schwesinger C., Licht C., Coward R.J., Fester L. (2020). Podocytes Produce and Secrete Functional Complement C3 and Complement Factor H. Front. Immunol..

[45] Li Y., Ni X., Li X., Kang Y., Yuan X., Xu G., Wang T., Li D., Shi S., Lv J. (2025). Glomerular mesangial cells derived complement factor H regulates complement activation, influences cell proliferation, and maintains actin cytoskeleton. Int. Immunopharmacol..

[46] Mr E., Verhasselt V., Kaashoek J.G., Timmerman J.J., Schroeijers W.E., Verweij C.L., van der Woude F.J., van Es L.A., Daha M.R. (1994). Regulation of C3 and factor H synthesis of human glomerular mesangial cells by IL-1 and interferon-gamma. Clin. Exp. Immunol..

[47] Montinaro V., Serra L., Perissutti S., Ranieri E., Tedesco F., Schena F.P. (1995). Biosynthesis of C3 by human mesangial cells. Modulation by proinflammatory cytokines. Kidney Int..

[48] Cosio F.G., Shibata T., Rovin B.H., Birmingham D.J. (1994). Effects of complement activation products on the synthesis of decay accelerating factor and membrane cofactor protein by human mesangial cells. Kidney Int..

[49] Brooimans R.A., Stegmann A.P., van Dorp W.T., van der Ark A.A., van der Woude F.J., van Es L.A., Daha M.R. (1991). Interleukin 2 mediates stimulation of complement C3 biosynthesis in human proximal tubular epithelial cells. J. Clin. Investig..

[50] Gerritsma J.S., Gerritsen A.F., Van Kooten C., Van Es L.A., Daha M.R. (1996). Interleukin-1 alpha enhances the biosynthesis of complement C3 and factor B by human kidney proximal tubular epithelial cells in vitro. Mol. Immunol..

[51] Welch T.R., Beischel L.S., Witte D.P. (1993). Differential expression of complement C3 and C4 in the human kidney. J. Clin. Investig..

[52] Portilla D., Sabapathy V., Chauss D. (2025). Role of local complement activation in kidney fibrosis and repair. J. Clin. Investig..

[53] Castellano G., Cappiello V., Fiore N., Pontrelli P., Gesualdo L., Schena F.P., Montinaro V. (2005). CD40 ligand increases complement C3 secretion by proximal tubular epithelial cells. J. Am. Soc. Nephrol..

[54] Sheerm N.S., Risley P., Abe K., Tang Z., Wong W., Lin T., Sacks S.H. (2007). Synthesis of complement protein C3 in the kidney is an important mediator of local tissue injury. FASEB J..

[55] Wendt R., Siwy J., He T., Latosinska A., Wiech T., Zipfel P.F., Tserga A., Vlahou A., Rupprecht H., Catanese L. (2021). Molecular Mapping of Urinary Complement Peptides in Kidney Diseases. Proteomes.

[56] Kesarwani V., Bukhari M.H., Kahlenberg J.M., Wang S. (2024). Urinary complement biomarkers in immune-mediated kidney diseases. Front. Immunol..

[57] Zhu G., Qiu W., Li Y., Zhao C., He F., Zhou M., Wang L., Zhao D., Lu Y., Zhang J. (2017). Sublytic C5b-9 Induces Glomerular Mesangial Cell Apoptosis through the Cascade Pathway of MEKK2–p38 MAPK–IRF-1–TRADD–Caspase 8 in Rat Thy-1 Nephritis. J. Immunol..

[58] Yao C., He F., Liu L., Zhang Z., Zhao C., Qiu W., Zhao D., Zhang J., Xie M., Gong Y. (2018). Sublytic C5b-9 Induces Glomerular Mesangial Cell Apoptosis Through miR-3546/SOX4/Survivin Axis in Rat Thy-1 Nephritis. Cell. Physiol. Biochem..

[59] Hila S., Soane L., Koski C.L. (2001). Sublytic C5b-9-stimulated Schwann cell survival through PI 3-kinase-mediated phosphorylation of BAD. Glia.

[60] Guo R., Wang Y., Minto A.W., Quigg R.J., Cunningham P.N. (2004). Acute renal failure in endotoxemia is dependent on caspase activation. J. Am. Soc. Nephrol..

[61] Linkermann A., Green D.R. (2014). Necroptosis. N. Engl. J. Med..

[62] Tonnus W., Linkermann A. (2017). The in vivo evidence for regulated necrosis. Immunol. Rev..

[63] Gupta A., Chakole S., Agrawal S., Khekade H., Prasad R., Lohakare T., Wanjari M. (2023). Emerging Insights Into Necroptosis: Implications for Renal Health and Diseases. Cureus.

[64] Guo Y., Zhou J., Wang Y., Wu X., Mou Y., Song X. (2024). Cell type-specific molecular mechanisms and implications of necroptosis in inflammatory respiratory diseases. Immunol. Rev..

[65] Vanden Berghe T., Hassannia B., Vandenabeele P. (2016). An outline of necrosome triggers. Cell. Mol. Life Sci..

[66] Degterev A., Maki J.L., Yuan J. (2013). Activity and specificity of necrostatin-1, small-molecule inhibitor of RIP1 kinase. Cell Death Differ..

[67] Yuk H., Abdullah M., Kim D.H., Lee H., Lee S.J. (2021). Necrostatin-1 Prevents Ferroptosis in a RIPK1- and IDO-Independent Manner in Hepatocellular Carcinoma. Antioxidants.

[68] Heckmann B.L., Tummers B., Green D.R. (2019). Crashing the computer: Apoptosis vs. necroptosis in neuroinflammation. Cell Death Differ..

[69] Tran H.T., Kratina T., Coutansais A., Michalek D., Hogan B.M., Lawlor K.E., Vince J.E., Silke J., Lalaoui N. (2024). RIPK3 cleavage is dispensable for necroptosis inhibition but restricts NLRP3 inflammasome activation. Cell Death Differ..

[70] Frank D., Vince J.E. (2019). Pyroptosis versus necroptosis: Similarities, differences, and crosstalk. Cell Death Differ..

[71] Belavgeni A., Meyer C., Stumpf J., Hugo C., Linkermann A. (2020). Ferroptosis and Necroptosis in the Kidney. Cell Chem. Biol..

[72] Yu Q., Chen Y., Zhao Y., Huang S., Xin X., Jiang L., Wang H., Wu W., Qu L., Xiang C. (2023). Nephropathy Is Aggravated by Fatty Acids in Diabetic Kidney Disease through Tubular Epithelial Cell Necroptosis and Is Alleviated by an RIPK-1 Inhibitor. Kidney Dis..

[73] Chen H., Fang Y., Wu J., Chen H., Zou Z., Zhang X., Shao J., Xu Y. (2018). RIPK3-MLKL-mediated necroinflammation contributes to AKI progression to CKD. Cell Death Dis..

[74] Mulay S.R., Desai J., Kumar S.V., Eberhard J.N., Thomasova D., Romoli S., Grigorescu M., Kulkarni O.P., Popper B., Vielhauer V. (2016). Cytotoxicity of crystals involves RIPK3-MLKL-mediated necroptosis. Nat. Commun..

[75] Schreiber A., Rousselle A., Becker J.U., von Massenhausen A., Linkermann A., Kettritz R. (2017). Necroptosis controls NET generation and mediates complement activation, endothelial damage, and autoimmune vasculitis. Proc. Natl. Acad. Sci. USA.

[76] Newton K., Dugger D.L., Maltzman A., Greve J.M., Hedehus M., Martin-McNulty B., Carano R.A., Cao T.C., van Bruggen N., Bernstein L. (2016). RIPK3 deficiency or catalytically inactive RIPK1 provides greater benefit than MLKL deficiency in mouse models of inflammation and tissue injury. Cell Death Differ..

[77] Lusthaus M., Mazkereth N., Donin N., Fishelson Z. (2018). Receptor-Interacting Protein Kinases 1 and 3, and Mixed Lineage Kinase Domain-Like Protein Are Activated by Sublytic Complement and Participate in Complement-Dependent Cytotoxicity. Front. Immunol..

[78] Jiang L., Zhao L., Liu Y., Fu Z., Wu M., Mou Z., Li M. (2025). PANoptosis: A novel therapeutic target in kidney disease (Review). Int. J. Mol. Med..

[79] Maremonti F., Meyer C., Linkermann A. (2022). Mechanisms and Models of Kidney Tubular Necrosis and Nephron Loss. J. Am. Soc. Nephrol..

[80] Zhou X.Y., Lin B., Chen W., Cao R.Q., Guo Y., Said A., Khan T., Zhang H.L., Zhu Y.M. (2023). The brain protection of MLKL inhibitor necrosulfonamide against focal ischemia/reperfusion injury associating with blocking the nucleus and nuclear envelope translocation of MLKL and RIP3K. Front. Pharmacol..

[81] Sugaya T., Kanno H., Matsuda M., Handa K., Tateda S., Murakami T., Ozawa H., Itoi E. (2019). B-RAF(V600E) Inhibitor Dabrafenib Attenuates RIPK3-Mediated Necroptosis and Promotes Functional Recovery after Spinal Cord Injury. Cells.

[82] Kearney C.J., Cullen S.P., Clancy D., Martin S.J. (2014). RIPK1 can function as an inhibitor rather than an initiator of RIPK3-dependent necroptosis. FEBS J..

[83] Hou B., Liu M., Chen Y., Ni W., Suo X., Xu Y., He Q., Meng X., Hao Z. (2022). Cpd-42 protects against calcium oxalate nephrocalcinosis-induced renal injury and inflammation by targeting RIPK3-mediated necroptosis. Front. Pharmacol..

[84] Wang J.N., Liu M.M., Wang F., Wei B., Yang Q., Cai Y.T., Chen X., Liu X.Q., Jiang L., Li C. (2019). RIPK1 inhibitor Cpd-71 attenuates renal dysfunction in cisplatin-treated mice via attenuating necroptosis, inflammation and oxidative stress. Clin. Sci..

[85] Linkermann A., Brasen J.H., Himmerkus N., Liu S., Huber T.B., Kunzendorf U., Krautwald S. (2012). Rip1 (receptor-interacting protein kinase 1) mediates necroptosis and contributes to renal ischemia/reperfusion injury. Kidney Int..

[86] Zhang X., Wang Q., Cao G., Luo M., Hou H., Yue C. (2023). Pyroptosis by NLRP3/caspase-1/gasdermin-D pathway in synovial tissues of rheumatoid arthritis patients. J. Cell Mol. Med..

[87] Ding J., Wang K., Liu W., She Y., Sun Q., Shi J., Sun H., Wang D.C., Shao F. (2016). Erratum: Pore-forming activity and structural autoinhibition of the gasdermin family. Nature.

[88] Shi J., Zhao Y., Wang K., Shi X., Wang Y., Huang H., Zhuang Y., Cai T., Wang F., Shao F. (2015). Cleavage of GSDMD by inflammatory caspases determines pyroptotic cell death. Nature.

[89] Russo A.J., Behl B., Banerjee I., Rathinam V.A.K. (2018). Emerging Insights into Noncanonical Inflammasome Recognition of Microbes. J. Mol. Biol..

[90] Ge Z., Xu J., Yang K., Wu L., Chen S., Chen B., Tian J., Zhang J., Xu A., Huang B. (2024). Molecular mechanism of bovine Gasdermin D-mediated pyroptosis. Vet. Res..

[91] Deng M., Scott M.J., Fan J., Billiar T.R. (2019). Location is the key to function: HMGB1 in sepsis and trauma-induced inflammation. J. Leukoc. Biol..

[92] Hempel A., D’Osualdo A., Snipas S.J., Salvesen G.S. (2023). Cell organelles are retained inside pyroptotic corpses during inflammatory cell death. Biosci. Rep..

[93] Yang D., He Y., Muñoz-Planillo R., Liu Q., Núñez G. (2015). Caspase-11 Requires the Pannexin-1 Channel and the Purinergic P2X7 Pore to Mediate Pyroptosis and Endotoxic Shock. Immunity.

[94] Ruhl S., Shkarina K., Demarco B., Heilig R., Santos J.C., Broz P. (2018). ESCRT-dependent membrane repair negatively regulates pyroptosis downstream of GSDMD activation. Science.

[95] Kayagaki N., Stowe I.B., Lee B.L., O’Rourke K., Anderson K., Warming S., Cuellar T., Haley B., Roose-Girma M., Phung Q.T. (2015). Caspase-11 cleaves gasdermin D for non-canonical inflammasome signalling. Nature.

[96] Wang Y., Gao W., Shi X., Ding J., Liu W., He H., Wang K., Shao F. (2017). Chemotherapy drugs induce pyroptosis through caspase-3 cleavage of a gasdermin. Nature.

[97] Tsuchiya K., Nakajima S., Hosojima S., Thi Nguyen D., Hattori T., Manh Le T., Hori O., Mahib M.R., Yamaguchi Y., Miura M. (2019). Caspase-1 initiates apoptosis in the absence of gasdermin D. Nat. Commun..

[98] Li Y., Xia W., Wu M., Yin J., Wang Q., Li S., Zhang A., Huang S., Zhang Y., Jia Z. (2020). Activation of GSDMD contributes to acute kidney injury induced by cisplatin. Am. J. Physiol. Ren. Physiol..

[99] Wu D., Ai L., Sun Y., Yang B., Chen S., Wang Q., Kuang H. (2021). Role of NLRP3 Inflammasome in Lupus Nephritis and Therapeutic Targeting by Phytochemicals. Front. Pharmacol..

[100] Chen F., Lu J., Yang X., Xiao B., Chen H., Pei W., Jin Y., Wang M., Li Y., Zhang J. (2020). Acetylbritannilactone attenuates contrast-induced acute kidney injury through its anti-pyroptosis effects. Biosci. Rep..

[101] Wang F., Huang M., Wang Y., Hong Y., Zang D., Yang C., Wu C., Zhu Q. (2022). Membrane Attack Complex C5b-9 Promotes Renal Tubular Epithelial Cell Pyroptosis in Trichloroethylene-Sensitized Mice. Front. Pharmacol..

[102] Xiong W., Meng X.-F., Zhang C. (2020). Inflammasome activation in podocytes: A new mechanism of glomerular diseases. Inflamm. Res..

[103] Islamuddin M., Qin X. (2024). Renal macrophages and NLRP3 inflammasomes in kidney diseases and therapeutics. Cell Death Discov..

[104] Cai Q., Sun Z., Xu S., Jiao X., Guo S., Li Y., Wu H., Yu X. (2022). Disulfiram ameliorates ischemia/reperfusion-induced acute kidney injury by suppressing the caspase-11-GSDMD pathway. Ren. Fail..

[105] Hutton H.L., Ooi J.D., Holdsworth S.R., Kitching A.R. (2016). The NLRP3 inflammasome in kidney disease and autoimmunity. Nephrology.

[106] Turner C.M., Arulkumaran N., Singer M., Unwin R.J., Tam F.W. (2014). Is the inflammasome a potential therapeutic target in renal disease?. BMC Nephrol..

[107] Stockwell B.R., Angeli J.P.F., Bayir H., Bush A.I., Conrad M., Dixon S.J., Fulda S., Gascón S., Hatzios S.K., Kagan V.E. (2017). Ferroptosis: A Regulated Cell Death Nexus Linking Metabolism, Redox Biology, and Disease. Cell.

[108] Xie Y., Hou W., Song X., Yu Y., Huang J., Sun X., Kang R., Tang D. (2016). Ferroptosis: Process and function. Cell Death Differ..

[109] Dixon S.J., Lemberg K.M., Lamprecht M.R., Skouta R., Zaitsev E.M., Gleason C.E., Patel D.N., Bauer A.J., Cantley A.M., Yang W.S. (2012). Ferroptosis: An iron-dependent form of nonapoptotic cell death. Cell.

[110] Kagan V.E., Mao G., Qu F., Angeli J.P., Doll S., Croix C.S., Dar H.H., Liu B., Tyurin V.A., Ritov V.B. (2017). Oxidized arachidonic and adrenic PEs navigate cells to ferroptosis. Nat. Chem. Biol..

[111] Dixon S.J., Winter G.E., Musavi L.S., Lee E.D., Snijder B., Rebsamen M., Superti-Furga G., Stockwell B.R. (2015). Human Haploid Cell Genetics Reveals Roles for Lipid Metabolism Genes in Nonapoptotic Cell Death. ACS Chem. Biol..

[112] Doll S., Proneth B., Tyurina Y.Y., Panzilius E., Kobayashi S., Ingold I., Irmler M., Beckers J., Aichler M., Walch A. (2017). ACSL4 dictates ferroptosis sensitivity by shaping cellular lipid composition. Nat. Chem. Biol..

[113] Bersuker K., Hendricks J.M., Li Z., Magtanong L., Ford B., Tang P.H., Roberts M.A., Tong B., Maimone T.J., Zoncu R. (2019). The CoQ oxidoreductase FSP1 acts parallel to GPX4 to inhibit ferroptosis. Nature.

[114] Wan C., Lin J.H., Hammes H.P., Zhang C. (2022). Mitochondrial Oxidative Stress and Cell Death in Podocytopathies. Biomolecules.

[115] Li Y., Li K., Zhao W., Wang H., Xue X., Chen X., Li W., Xu P., Wang K., Liu P. (2023). VPA improves ferroptosis in tubular epithelial cells after cisplatin-induced acute kidney injury. Front. Pharmacol..

[116] Zhang X., Li X. (2022). Abnormal Iron and Lipid Metabolism Mediated Ferroptosis in Kidney Diseases and Its Therapeutic Potential. Metabolites.

[117] Feng X., Wang S., Sun Z., Dong H., Yu H., Huang M., Gao X. (2021). Ferroptosis Enhanced Diabetic Renal Tubular Injury via HIF-1alpha/HO-1 Pathway in db/db Mice. Front. Endocrinol..

[118] Long Z., Luo Y., Yu M., Wang X., Zeng L., Yang K. (2024). Targeting ferroptosis: A new therapeutic opportunity for kidney diseases. Front. Immunol..

[119] Liu Z., Ma J., Zuo X., Zhang X., Hong Y., Cai S., Huang H., Wang F., Wu C., Zhang J. (2022). C5b-9 mediates ferroptosis of tubular epithelial cells in trichloroethylene-sensitization mice. Ecotoxicol. Environ. Saf..

[120] Xia C., Sun X., Shao J., Min J., Wei C., Zhao F., Fu C., Zhang Q. (2026). Selenomethionine as a dual-mechanism ferroptosis inhibitor: Selenium-supply-driven GPX4 biosynthesis beyond transsulfuration and reductive-capacity-mediated ROS scavenging independent of GPX4 activity. Cell Death Dis..

[121] Kim J., Kundu M., Viollet B., Guan K.L. (2011). AMPK and mTOR regulate autophagy through direct phosphorylation of Ulk1. Nat. Cell Biol..

[122] Lee J.W., Park S., Takahashi Y., Wang H.G. (2010). The association of AMPK with ULK1 regulates autophagy. PLoS ONE.

[123] Wong P.M., Puente C., Ganley I.G., Jiang X. (2013). The ULK1 complex: Sensing nutrient signals for autophagy activation. Autophagy.

[124] Zhao Y.G., Codogno P., Zhang H. (2021). Machinery, regulation and pathophysiological implications of autophagosome maturation. Nat. Rev. Mol. Cell Biol..

[125] Ryter S.W., Mizumura K., Choi A.M. (2014). The impact of autophagy on cell death modalities. Int. J. Cell Biol..

[126] Su Z., Yang Z., Xu Y., Chen Y., Yu Q. (2015). Apoptosis, autophagy, necroptosis, and cancer metastasis. Mol. Cancer.

[127] Wu Z., Deng J., Zhou H., Tan W., Lin L., Yang J. (2022). Programmed Cell Death in Sepsis Associated Acute Kidney Injury. Front. Med..

[128] Sorbara M.T., Foerster E.G., Tsalikis J., Abdel-Nour M., Mangiapane J., Sirluck-Schroeder I., Tattoli I., van Dalen R., Isenman D.E., Rohde J.R. (2018). Complement C3 Drives Autophagy-Dependent Restriction of Cyto-invasive Bacteria. Cell Host Microbe.

[129] King B.C., Kulak K., Colineau L., Blom A.M. (2021). Outside in: Roles of complement in autophagy. Br. J. Pharmacol..

[130] Am C., Kulak K., Krus U., Rosberg R., Golec E., Wozniak K., Gomez M.F., Zhang E., O’Connell D.J., Renström E. (2019). Complement Component C3 Is Highly Expressed in Human Pancreatic Islets and Prevents β Cell Death via ATG16L1 Interaction and Autophagy Regulation. Cell Metab..

[131] King B.C., Blom A.M. (2023). Intracellular complement: Evidence, definitions, controversies, and solutions. Immunol. Rev..

[132] Tang C., Livingston M.J., Liu Z., Dong Z. (2020). Autophagy in kidney homeostasis and disease. Nat. Rev. Nephrol..

[133] Lin T.A., Wu V.C., Wang C.Y. (2019). Autophagy in Chronic Kidney Diseases. Cells.

[134] Hartleben B., Godel M., Meyer-Schwesinger C., Liu S., Ulrich T., Kobler S., Wiech T., Grahammer F., Arnold S.J., Lindenmeyer M.T. (2010). Autophagy influences glomerular disease susceptibility and maintains podocyte homeostasis in aging mice. J. Clin. Investig..

[135] Lenoir O., Jasiek M., Henique C., Guyonnet L., Hartleben B., Bork T., Chipont A., Flosseau K., Bensaada I., Schmitt A. (2015). Endothelial cell and podocyte autophagy synergistically protect from diabetes-induced glomerulosclerosis. Autophagy.

[136] Lin Q., Banu K., Ni Z., Leventhal J.S., Menon M.C. (2021). Podocyte Autophagy in Homeostasis and Disease. J. Clin. Med..

[137] Livingston M.J., Ding H.F., Huang S., Hill J.A., Yin X.M., Dong Z. (2016). Persistent activation of autophagy in kidney tubular cells promotes renal interstitial fibrosis during unilateral ureteral obstruction. Autophagy.

[138] Xu Y., Ruan S., Wu X., Chen H., Zheng K., Fu B. (2013). Autophagy and apoptosis in tubular cells following unilateral ureteral obstruction are associated with mitochondrial oxidative stress. Int. J. Mol. Med..

[139] Ding Y., Kim S., Lee S.Y., Koo J.K., Wang Z., Choi M.E. (2014). Autophagy regulates TGF-beta expression and suppresses kidney fibrosis induced by unilateral ureteral obstruction. J. Am. Soc. Nephrol..

[140] Ma M., Pan Y., Zhang Y., Yang M., Xi Y., Lin B., Hao W., Liu J., Wu L., Liu Y. (2023). Metformin combined with rapamycin ameliorates podocyte injury in idiopathic membranous nephropathy through the AMPK/mTOR signaling pathway. J. Cell Commun. Signal..

[141] Hu X.M., Li Z.X., Lin R.H., Shan J.Q., Yu Q.W., Wang R.X., Liao L.S., Yan W.T., Wang Z., Shang L. (2021). Guidelines for Regulated Cell Death Assays: A Systematic Summary, A Categorical Comparison, A Prospective. Front. Cell Dev. Biol..

[142] Hughes J., Gobe G. (2007). Identification and quantification of apoptosis in the kidney using morphology, biochemical and molecular markers. Nephrology.

[143] Liu X., Lieberman J. (2020). Knocking ’em Dead: Pore-Forming Proteins in Immune Defense. Annu. Rev. Immunol..

[144] von Massenhausen A., Tonnus W., Himmerkus N., Parmentier S., Saleh D., Rodriguez D., Ousingsawat J., Ang R.L., Weinberg J.M., Sanz A.B. (2018). Phenytoin inhibits necroptosis. Cell Death Dis..

[145] Tonnus W., Meyer C., Paliege A., Belavgeni A., von Massenhausen A., Bornstein S.R., Hugo C., Becker J.U., Linkermann A. (2019). The pathological features of regulated necrosis. J. Pathol..

[146] He W.T., Wan H., Hu L., Chen P., Wang X., Huang Z., Yang Z.H., Zhong C.Q., Han J. (2015). Gasdermin D is an executor of pyroptosis and required for interleukin-1beta secretion. Cell Res..

[147] Guey B., Bodnar M., Manie S.N., Tardivel A., Petrilli V. (2014). Caspase-1 autoproteolysis is differentially required for NLRP1b and NLRP3 inflammasome function. Proc. Natl. Acad. Sci. USA.

[148] Yoshida M., Minagawa S., Araya J., Sakamoto T., Hara H., Tsubouchi K., Hosaka Y., Ichikawa A., Saito N., Kadota T. (2019). Involvement of cigarette smoke-induced epithelial cell ferroptosis in COPD pathogenesis. Nat. Commun..

[149] Thi Nghiem T.H., Kusuma F., Park J., Joe Y., Chung H.T., Han J. (2025). Brief guide to detecting ferroptosis. Mol. Cells.

[150] Klionsky D.J., Abdel-Aziz A.K., Abdelfatah S., Abdellatif M., Abdoli A., Abel S., Abeliovich H., Abildgaard M.H., Abudu Y.P., Acevedo-Arozena A. (2021). Guidelines for the use and interpretation of assays for monitoring autophagy (4th edition)(1). Autophagy.

[151] Mizushima N., Yoshimori T., Levine B. (2010). Methods in mammalian autophagy research. Cell.

[152] Lee Y.K., Jun Y.W., Choi H.E., Huh Y.H., Kaang B.K., Jang D.J., Lee J.A. (2017). Development of LC3/GABARAP sensors containing a LIR and a hydrophobic domain to monitor autophagy. EMBO J..

[153] Matos Silva G., Venda J., Afonso N., Leal R., Cerqueira S., Galvão A., Sá (2026). Complement role in kidney disease: A comprehensive review and therapeutic innovations. BMC Nephrol..

[154] Konieczny A., Villa L., Schult A.L., Bücher E., Rong S., Kunter U., van Roeyen C.R., Polakowski T., Hawlisch H., Hillebrandt S. (2007). Complement C5 mediates experimental tubulointerstitial fibrosis. J. Am. Soc. Nephrol. JASN.

[155] Beck L., Debiec H., Fervenza F.C., Hou F.F., Jha V., Sethi S., Tong A., Vivarelli M., Wetzels J. (2021). Membranous nephropathy. Nat. Rev. Dis. Primers.

[156] Kistler A.D., Salant D.J. (2024). Complement activation and effector pathways in membranous nephropathy. Kidney Int..

[157] Seifert L., Zahner G., Meyer-Schwesinger C., Hickstein N., Dehde S., Wulf S., Kollner S.M.S., Lucas R., Kylies D., Froembling S. (2023). The classical pathway triggers pathogenic complement activation in membranous nephropathy. Nat. Commun..

[158] Lehman A., Albawardi A., Satoskar A., Brodsky S., Nadasdy G., Hebert L., Rovin B., Nadasdy T. (2013). IgG subclass staining in renal biopsies with membranous glomerulonephritis indicates subclass switch during disease progression. Mod. Pathol..

[159] Shi X., Shi Y., Chang M., Duan H., Chen H., Tuerxun X., Zhao M., Shi Z., Zhang Y. (2026). Inhibition of ferroptosis exerts renal protective effects in membranous nephropathy rats via the Nrf2/HO-1 pathway. Eur. J. Pharmacol..

[160] Shi X., Zhang Q., Chang M., Zhang Y., Zhao M., Yang B., Li P., Zhang Y. (2023). Ferroptosis is involved in passive Heymann nephritis in rats. Heliyon.

[161] Gao S., Cui Z., Zhao M.-H. (2022). Complement C3a and C3a Receptor Activation Mediates Podocyte Injuries in the Mechanism of Primary Membranous Nephropathy. J. Am. Soc. Nephrol. JASN.

[162] Arrizabalaga P., Solé M., Iglesias C., Escaramís G., Ascaso C. (2006). Renal expression of ICAM-1 and VCAM-1 in ANCA-associated glomerulonephritis--are there differences among serologic subgroups?. Clin. Nephrol..

[163] Fervenza F.C., Appel G.B., Barbour S.J., Rovin B.H., Lafayette R.A., Aslam N., Jefferson J.A., Gipson P.E., Rizk D.V., Sedor J.R. (2019). Rituximab or Cyclosporine in the Treatment of Membranous Nephropathy. N. Engl. J. Med..

[164] Zhang Y., Yan X., Zhao T., Xu Q., Peng Q., Hu R., Quan S., Zhou Y., Xing G. (2017). Targeting C3a/C5a receptors inhibits human mesangial cell proliferation and alleviates immunoglobulin A nephropathy in mice. Clin. Exp. Immunol..

[165] Zhu L., Zhai Y.L., Wang F.M., Hou P., Lv J.C., Xu D.M., Shi S.F., Liu L.J., Yu F., Zhao M.H. (2015). Variants in Complement Factor H and Complement Factor H-Related Protein Genes, CFHR3 and CFHR1, Affect Complement Activation in IgA Nephropathy. J. Am. Soc. Nephrol..

[166] Qiu W., Zhang Y., Liu X., Zhou J., Li Y., Zhou Y., Shan K., Xia M., Che N., Feng X. (2012). Sublytic C5b-9 complexes induce proliferative changes of glomerular mesangial cells in rat Thy-1 nephritis through TRAF6-mediated PI3K-dependent Akt1 activation. J. Pathol..

[167] Xie M., Wu Z., Ying S., Liu L., Zhao C., Yao C., Zhang Z., Luo C., Wang W., Zhao D. (2021). Sublytic C5b-9 induces glomerular mesangial cell proliferation via ERK1/2-dependent SOX9 phosphorylation and acetylation by enhancing Cyclin D1 in rat Thy-1 nephritis. Exp. Mol. Med..

[168] Tang Z., Lu B., Hatch E., Sacks S.H., Sheerin N.S. (2026). C3a Mediates Epithelial-to-Mesenchymal Transition in Proteinuric Nephropathy. J. Am. Soc. Nephrol. JASN.

[169] Perkovic V., Barratt J., Rovin B., Kashihara N., Maes B., Zhang H., Trimarchi H., Kollins D., Papachristofi O., Jacinto-Sanders S. (2025). Alternative Complement Pathway Inhibition with Iptacopan in IgA Nephropathy. N. Engl. J. Med..

[170] Lafayette R.A., Rovin B.H., Reich H.N., Tumlin J.A., Floege J., Barratt J. (2020). Safety, Tolerability and Efficacy of Narsoplimab, a Novel MASP-2 Inhibitor for the Treatment of IgA Nephropathy. Kidney Int. Rep..

[171] Lafayette R., Tumlin J., Fenoglio R., Kaufeld J., Perez Valdivia M.A., Wu M.S., Susan Huang S.H., Alamartine E., Kim S.G., Yee M. (2025). Efficacy and Safety of Ravulizumab in IgA Nephropathy: A Phase 2 Randomized Double-Blind Placebo-Controlled Trial. J. Am. Soc. Nephrol..

[172] Barratt J., Liew A., Trimarchi H., Bruchfeld A., Heerspink H.J.L., Inker L., Zhang H., Radhakrishnan J., Schneider B., Marsala J. (2026). Ravulizumab in IgA nephropathy: A prespecified interim analysis of a randomized Phase 3 trial (I CAN). GlomCon Hawaii 2026 Abstracts. Rare Kidney Diseases.

[173] Barbour S.J., Hladunewich M.A., McCaleb M.L., Robson R., Barrett T.D., Yin L., Frazer-Abel A., Garg J.P., Geary R., Schneider E. (2026). A single-arm phase 2 trial of an investigational RNA therapeutic to complement factor B sefaxersen for treatment of IgA nephropathy. Kidney Int..

[174] Xiao H., Heeringa P., Hu P., Liu Z., Zhao M., Aratani Y., Maeda N., Falk R.J., Jennette J.C. (2002). Antineutrophil cytoplasmic autoantibodies specific for myeloperoxidase cause glomerulonephritis and vasculitis in mice. J. Clin. Investig..

[175] Xiao H., Schreiber A., Heeringa P., Falk R.J., Jennette J.C. (2007). Alternative complement pathway in the pathogenesis of disease mediated by anti-neutrophil cytoplasmic autoantibodies. Am. J. Pathol..

[176] Schreiber A., Xiao H., Jennette J.C., Schneider W., Luft F.C., Kettritz R. (2009). C5a receptor mediates neutrophil activation and ANCA-induced glomerulonephritis. J. Am. Soc. Nephrol. JASN.

[177] Dick J., Gan P.-Y., Ford S.L., Odobasic D., Alikhan M.A., Loosen S.H., Hall P., Westhorpe C.L., Li A., Ooi J.D. (2018). C5a receptor 1 promotes autoimmunity, neutrophil dysfunction and injury in experimental anti-myeloperoxidase glomerulonephritis. Kidney Int..

[178] Wang H., Wang C., Zhao M.-H., Chen M. (2015). Neutrophil extracellular traps can activate alternative complement pathways. Clin. Exp. Immunol..

[179] Yuen J., Pluthero F.G., Douda D.N., Riedl M., Cherry A., Ulanova M., Kahr W.H.A., Palaniyar N., Licht C. (2016). NETosing Neutrophils Activate Complement Both on Their Own NETs and Bacteria via Alternative and Non-alternative Pathways. Front. Immunol..

[180] Yousefi S., Mihalache C., Kozlowski E., Schmid I., Simon H.U. (2009). Viable neutrophils release mitochondrial DNA to form neutrophil extracellular traps. Cell Death Differ..

[181] Shiratori-Aso S., Nakazawa D. (2023). The involvement of NETs in ANCA-associated vasculitis. Front. Immunol..

[182] Murakami M., Aratani Y., Ishizu A., Atsumi T., Nakazawa D., Watanabe-Kusunoki K., Kanda M., Shiratori-Aso S., Abe N., Nishio S. (2023). Regulation of NETosis and Inflammation by Cyclophilin D in Myeloperoxidase-Positive Antineutrophil Cytoplasmic Antibody-Associated Vasculitis. Arthritis Rheumatol..

[183] Hao J., Chen M., Zhao M.-H. (2013). Involvement of protein kinase C in C5a-primed neutrophils for ANCA-mediated activation. Mol. Immunol..

[184] Hao J., Meng L.-Q., Xu P.-C., Chen M., Zhao M.-H. (2012). P38MAPK, ERK and PI3K signaling pathways are involved in C5a-primed neutrophils for ANCA-mediated activation. PLoS ONE.

[185] Hao J., Huang Y.-M., Zhao M.-H., Chen M. (2014). The interaction between C5a and sphingosine-1-phosphate in neutrophils for antineutrophil cytoplasmic antibody mediated activation. Arthritis Res. Ther..

[186] Halbwachs L., Lesavre P. (2012). Endothelium-neutrophil interactions in ANCA-associated diseases. J. Am. Soc. Nephrol. JASN.

[187] Gou S.-J., Yuan J., Wang C., Zhao M.-H., Chen M. (2013). Alternative complement pathway activation products in urine and kidneys of patients with ANCA-associated GN. Clin. J. Am. Soc. Nephrol. CJASN.

[188] Chen M., Xing G.-Q., Yu F., Liu G., Zhao M.-H. (2009). Complement deposition in renal histopathology of patients with ANCA-associated pauci-immune glomerulonephritis. Nephrol. Dial. Transplant..

[189] van Paassen P., van Rie H., Bijnens N., Heerings-Rewinkel P., van Breda Vriesman P., Cohen Tervaert J.W. (2017). Complement in ANCA-associated glomerulonephritis. Nephrol. Dial. Transplant..

[190] Chen S.-F., Wang F.-M., Li Z.-Y., Yu F., Zhao M.-H., Chen M. (2015). Plasma complement factor H is associated with disease activity of patients with ANCA-associated vasculitis. Arthritis Res. Ther..

[191] Chen S.-F., Wang F.-M., Li Z.-Y., Yu F., Chen M., Zhao M.-H. (2017). The functional activities of complement factor H are impaired in patients with ANCA-positive vasculitis. Clin. Immunol..

[192] Juto A., Martin M., Björk A., Padyukov L., Grönwall C., Antovic A., Bruchfeld A., Gunnarsson I., Blom A.M. (2025). Association of C4d with disease activity in anti-neutrophil cytoplasmic antibody-associated vasculitis: Evidence for classical/lectin complement pathway activation. Arthritis Res. Ther..

[193] Tampe D., Korsten P., Ströbel P., Tampe (2021). Complement Components C3 and C4 Indicate Vasculitis Manifestations to Distinct Renal Compartments in ANCA-Associated Glomerulonephritis. Int. J. Mol. Sci..

[194] European Medicines Agency Tavneos—Referral. EMA Recommends Revoking Marketing Authorisation for Tavneos. https://www.ema.europa.eu/en/medicines/human/referrals/tavneos.

[195] Dal Pozzolo R., Iorio L., Davanzo F., Zanatta E., Iaccarino L., Ramonda R., Gerli R., Doria A., Cafaro G., Padoan R. (2026). Evolving Therapeutic Strategies in ANCA-Associated Vasculitis: Current Standards and Emerging Targets for GPA and MPA. Clin. Rev. Allergy Immunol..

[196] Yoshida M., Sasaki M., Sugisaki K., Yamaguchi Y., Yamada M. (2013). Neutrophil extracellular trap components in fibrinoid necrosis of the kidney with myeloperoxidase-ANCA-associated vasculitis. Clin. Kidney J..

[197] Bao L., Cunningham P.N., Quigg R.J. (2015). Complement in Lupus Nephritis: New Perspectives. Kidney Dis..

[198] Davidson A. (2016). What is damaging the kidney in lupus nephritis? Nature reviews. Rheumatology.

[199] Parikh S.V., Almaani S., Brodsky S., Rovin B.H. (2020). Update on Lupus Nephritis: Core Curriculum 2020. Am. J. Kidney Dis..

[200] Vigne J., Meuleman M.S., Amen A., Rossi V., Dumestre P. (2025). Complement in systemic lupus erythematosus across time and space: From tolerance to tissue injury and from extracellular to intracellular functions. Curr. Opin. Immunol..

[201] Mc R., Medjeral-Thomas N.R., Gilmore A.C., Trivedi P., Seyb K., Farzaneh-Far R., Gunnarsson I., Zickert A., Cairns T.D., Lightstone L. (2019). Glomerular membrane attack complex is not a reliable marker of ongoing C5 activation in lupus nephritis. Kidney Int..

[202] Song D., Guo W.-Y., Wang F.-M., Li Y.-Z., Song Y., Yu F., Zhao M.-H. (2017). Complement Alternative Pathway׳s Activation in Patients With Lupus Nephritis. Am. J. Med. Sci..

[203] Bao L., Osawe I., Puri T., Lambris J.D., Haas M., Quigg R.J. (2005). C5a promotes development of experimental lupus nephritis which can be blocked with a specific receptor antagonist. Eur. J. Immunol..

[204] Mistry P., Kaplan M.J. (2017). Cell death in the pathogenesis of systemic lupus erythematosus and lupus nephritis. Clin. Immunol..

[205] Miao N., Wang Z., Wang Q., Xie H., Yang N., Wang Y., Wang J., Kang H., Bai W., Wang Y. (2023). Oxidized mitochondrial DNA induces gasdermin D oligomerization in systemic lupus erythematosus. Nat. Commun..

[206] Alexander J.J., Chaves L., Chang A., Quigg R.J. (2012). The C5a receptor has a key role in immune complex glomerulonephritis in complement factor H-deficient mice. Kidney Int..

[207] Thakare S.B., So P.N., Rodriguez S., Hassanein M., Lerma E., Wiegley N., GlomCon Editorial T. (2023). Novel Therapeutics for Management of Lupus Nephritis: What Is Next?. Kidney Med..

[208] Liszewski M.K., Elvington M., Kulkarni H.S., Atkinson J.P. (2017). Complement’s hidden arsenal: New insights and novel functions inside the cell. Mol. Immunol..

[209] Endale H.T., Tesfaye W., Mengstie T.A. (2023). ROS induced lipid peroxidation and their role in ferroptosis. Front. Cell Dev. Biol..

[210] Richards R.L., Gewurz H., Osmand A.P., Alving C.R. (1977). Interactions of C-reactive protein and complement with liposomes. Proc. Natl. Acad. Sci. USA.

[211] Joubert P.E., Meiffren G., Gregoire I.P., Pontini G., Richetta C., Flacher M., Azocar O., Vidalain P.O., Vidal M., Lotteau V. (2009). Autophagy induction by the pathogen receptor CD46. Cell Host Microbe.

[212] Fritsch M., Gunther S.D., Schwarzer R., Albert M.C., Schorn F., Werthenbach J.P., Schiffmann L.M., Stair N., Stocks H., Seeger J.M. (2019). Caspase-8 is the molecular switch for apoptosis, necroptosis and pyroptosis. Nature.

[213] Man S.M., Kanneganti T.D. (2016). Converging roles of caspases in inflammasome activation, cell death and innate immunity. Nat. Rev. Immunol..

[214] Lawlor K.E., Khan N., Mildenhall A., Gerlic M., Croker B.A., D’Cruz A.A., Hall C., Kaur Spall S., Anderton H., Masters S.L. (2015). RIPK3 promotes cell death and NLRP3 inflammasome activation in the absence of MLKL. Nat. Commun..

[215] Conos S.A., Chen K.W., De Nardo D., Hara H., Whitehead L., Nunez G., Masters S.L., Murphy J.M., Schroder K., Vaux D.L. (2017). Active MLKL triggers the NLRP3 inflammasome in a cell-intrinsic manner. Proc. Natl. Acad. Sci. USA.

[216] Legendre C.M., Licht C., Muus P., Greenbaum L.A., Babu S., Bedrosian C., Bingham C., Cohen D.J., Delmas Y., Douglas K. (2013). Terminal complement inhibitor eculizumab in atypical hemolytic-uremic syndrome. N. Engl. J. Med..

[217] (2023). Ravulizumab (Ultomiris): CADTH Reimbursement Recommendation: Indication: For the Treatment of Adult and Pediatric Patients 1 Month of Age and Older with Atypical Hemolytic Uremic Syndrome to Inhibit Complement-Mediated Thrombotic Microangiopathy.

[218] Barratt J., Eren N., Kashihara N., Maes B., Rizk D.V., Rovin B., Trimarchi H., Zhang H., Wang W., Kocyigit I. (2026). Iptacopan in IgA Nephropathy-Final 24-Month Data. N. Engl. J. Med..

[219] Furie R.A., Rovin B.H., Garg J.P., Santiago M.B., Aroca-Martinez G., Zuta Santillan A.E., Alvarez D., Navarro Sandoval C., Lila A.M., Tumlin J.A. (2025). Efficacy and Safety of Obinutuzumab in Active Lupus Nephritis. N. Engl. J. Med..

[220] Furie R., Rovin B.H., Houssiau F., Malvar A., Teng Y.K.O., Contreras G., Amoura Z., Yu X., Mok C.C., Santiago M.B. (2020). Two-Year, Randomized, Controlled Trial of Belimumab in Lupus Nephritis. N. Engl. J. Med..

[221] Rovin B.H., Teng Y.K.O., Ginzler E.M., Arriens C., Caster D.J., Romero-Diaz J., Gibson K., Kaplan J., Lisk L., Navarra S. (2021). Efficacy and safety of voclosporin versus placebo for lupus nephritis (AURORA 1): A double-blind, randomised, multicentre, placebo-controlled, phase 3 trial. Lancet.

[222] Uhlin F., Szpirt W., Kronbichler A., Bruchfeld A., Soveri I., Rostaing L., Daugas E., Lionet A., Kamar N., Rafat C. (2022). Endopeptidase Cleavage of Anti-Glomerular Basement Membrane Antibodies in vivo in Severe Kidney Disease: An Open-Label Phase 2a Study. J. Am. Soc. Nephrol..

